# Non-Contrast and Contrast-Enhanced Cardiac Computed Tomography Imaging in the Diagnostic and Prognostic Evaluation of Coronary Artery Disease

**DOI:** 10.3390/diagnostics13122074

**Published:** 2023-06-15

**Authors:** Luca Pugliese, Francesca Ricci, Giacomo Sica, Mariano Scaglione, Salvatore Masala

**Affiliations:** 1 Radiology Unit, Department of Medical-Surgical Sciences and Translational Medicine, Sapienza University of Rome, Sant’Andrea University Hospital, 00189 Rome, Italy; l.pugliese88@gmail.com; 2 Radiology Unit, Department of Medical, Surgical and Experimental Sciences, University of Sassari, 07100 Sassari, Italy; fraricci891@gmail.com (F.R.); mscaglione@uniss.it (M.S.); 3 Radiology Unit, Monaldi Hospital, 80131 Napoli, Italy; gsica@sirm.org

**Keywords:** cardiac computed tomography, coronary computed tomography angiography, coronary artery calcium, epicardial adipose tissue, coronary stenosis, myocardial ischemia, fractional flow reserve, plaque composition, high-risk plaque features, perivascular adipose tissue

## Abstract

In recent decades, cardiac computed tomography (CT) has emerged as a powerful non-invasive tool for risk stratification, as well as the detection and characterization of coronary artery disease (CAD), which remains the main cause of morbidity and mortality in the world. Advances in technology have favored the increasing use of cardiac CT by allowing better performance with lower radiation doses. Coronary artery calcium, as assessed by non-contrast CT, is considered to be the best marker of subclinical atherosclerosis, and its use is recommended for the refinement of risk assessment in low-to-intermediate risk individuals. In addition, coronary CT angiography (CCTA) has become a gate-keeper to invasive coronary angiography (ICA) and revascularization in patients with acute chest pain by allowing the assessment not only of the extent of lumen stenosis, but also of its hemodynamic significance if combined with the measurement of fractional flow reserve or perfusion imaging. Moreover, CCTA provides a unique incremental value over functional testing and ICA by imaging the vessel wall, thus allowing the assessment of plaque burden, composition, and instability features, in addition to perivascular adipose tissue attenuation, which is a marker of vascular inflammation. There exists the potential to identify the non-obstructive lesions at high risk of progression to plaque rupture by combining all of these measures.

## 1. Introduction

Coronary artery disease (CAD) remains the main cause of morbidity and mortality in the world. The American Heart Association 2022 statistical update reported a high prevalence and incidence of CAD [1], which affects 20.1 million Americans ≥20 years of age and is estimated to occur in 720,000 and 335,000 individuals as a new or recurrent (fatal or non-fatal) event, respectively. The same source reports a decreasing trend in morbidity and mortality from CAD, which has occurred despite a worsening in the risk profiles of Americans with atherosclerotic cardiovascular disease (ASCVD), [2] and is likely due to advances in the prediction, detection, and treatment of CAD.

Historically, the prediction of CAD has been based on traditional cardiovascular risk factors and algorithms incorporating them, which provide an estimate of the risk of developing fatal and/or non-fatal coronary and other ASCVD events. Unfortunately, the performance of prediction algorithms is insufficient at the individual level for several reasons, such as a lack of validation in external cohorts or different populations and the lack of regular updates with contemporary epidemiological data [3]. Furthermore, relevant variables may not have been included in these models, prompting the addition of non-traditional cardiovascular risk factors and/or measures of subclinical ASCVD [4].

Instead, the detection of CAD has been based on the presence of symptoms combined with the demonstration of myocardial ischemia/dysfunction and arterial stenosis by stress (functional) testing and invasive coronary angiography (ICA), respectively. However, symptoms, including chest pain and anginal equivalents, such as dyspnea, diaphoresis, fatigue, and non-chest pain, have limited sensitivity and specificity [5], and may even be absent (silent angina) [6], especially in diabetic individuals [7]. Similarly, functional tests, including stress electrocardiography (ECG), echocardiography, and nuclear myocardial perfusion imaging (MPI), yield an insufficient diagnostic accuracy for detecting obstructive CAD in terms of both sensitivity and specificity [8]. Finally, elective ICA, which is considered the gold standard for CAD diagnosis, provides a two-dimensional “lumenogram” of the coronary arteries, but not images of the vessel wall or information on the hemodynamic consequences of stenoses [9], unless it is combined with the assessment of invasive fractional flow reserve (FFR), the use of which is increasing but still limited [10]. Furthermore, the diagnostic yield is low, as only slightly more than one third of patients with suspected CAD were found to have obstructive lesions upon ICA [11].

In recent decades, cardiac computed tomography (CT) has been increasingly recognized as a powerful non-invasive tool for the diagnostic and prognostic evaluation of CAD, and also due to advances in technology that allowed better performance with lower radiation doses [12]. Robust evidence is now available supporting the incorporation of cardiac CT in current CAD guidelines. This article briefly reviews the role of cardiac imaging by non-contrast and contrast enhanced CT in CAD evaluation, in addition to the positioning of these procedures in the diagnostic and prognostic flow chart.

## 2. Non-Contrast CT

Non-contrast CT is currently used for the assessment of coronary artery calcium (CAC), which is considered to be the best marker of subclinical CAD/ASCVD [13]. In addition, it can provide important information with regard to epicardial adipose tissue (EAT), which is a marker of systemic inflammation and ASCVD risk.

### 2.1. Coronary Artery Calcium

Intimal calcification has long been recognized as a typical feature of atherosclerotic lesions, with its extent increasing in parallel with the progression of vascular pathology toward the advanced stage [14]. It starts as microcalcification nuclei originating from vascular smooth muscle cell (VSMC)-derived apoptotic bodies and macrophage-derived matrix vesicles [15] in close association with inflammation [16]. With the progression of atherosclerosis, calcium deposits increase in size and become visible upon imaging as spotty calcification, along with increased plaque instability and risk of rupture [17,18]. Conversely, in more advanced stages, the coalescence of calcium deposits into large, sheet-like plates is related to the blunting of inflammation, which allows for the survival of VSMCs that produce collagen and undergo osteogenic differentiation [17,18]. The transition to uninflamed, fibrocalcific lesions is associated with plaque stabilization [18], unless calcified plates fracture and form noduli that protrude into the lumen, an event which is, however, uncommon in the coronary arteries [19]. Thus, the relationship between plaque instability and the extent of calcification appears to be non-linear, since the risk of rupture is low with no calcification, increases progressively with mild and moderate calcification, and decreases with severe calcification [15].

Non-contrast CT is increasingly used for assessing CAC and quantifying it using scoring systems such as the Agatston score, which is determined by the product of the calcified plaque area and the maximal calcium lesion density (from 1 to 4 based on Hounsfield units) [20]. Several studies have shown that the CAC score is a powerful predictor of morbidity and mortality from CAD and other ASCVDs.

The prognostic value of the CAC score was first demonstrated in asymptomatic individuals [21]. In the Multi-Ethnic Study of Atherosclerosis (MESA), which included participants 45 to 84 years of age, the overall CAC prevalence ranged from 52.1% to 70.4% in males, and from 34.6% to 44.6%, in females, depending on the ethnicity [22]. In this cohort, the adjusted risk of a coronary event was increased 7.73-fold in participants with CAC scores between 101 and 300, and 9.67-fold in those with CAC scores >300, as compared with those with no CAC; moreover, a doubling of the CAC score increased the risk of a major coronary event by 15 to 35%, and the risk of any coronary event by 18 to 39% [23]. The prevalence of CAC was shown to be lower in the younger participants in the Coronary Artery Risk Development in Young Adults (CARDIA), i.e., 5.5% among those aged 33 to 39 years, and 13.3% among those aged 40 to 45 years [24]. However, even in this younger cohort, the adjusted risk of coronary events was increased five-fold among participants with any CAC and 2.6-, 5.8-, and 9.8-fold among those with CAC scores of 1–19, 20–99, and >100, respectively [25]. Furthermore, CAC progression was found to correlate with the progression of all types of coronary plaque, including non-calcified plaques, whereas no plaque progression was observed in individuals with no CAC progression [26]. The prognostic value of the CAC score for CAD events in asymptomatic individuals has recently been confirmed by a systematic review of 45 studies [27].

Several other studies have subsequently showed that the CAC score improves risk stratification when added to algorithms based on traditional ASCVD risk factors [28], especially among individuals at intermediate risk, such as those with a Framingham Risk Score of 10–20% or an Adult Treatment Panel score of 6–20%, with reclassification to the high-risk category [29,30]. Moreover, the addition of the CAC score to a cardiovascular risk factor-based algorithm developed in the MESA cohort significantly improved risk prediction and performed well in the external validation cohorts of the Heinz Nixdorf Recall (HNR) Study and the Dallas Heart Study (DHS) [31]. In women, who were shown to have a lower prevalence of CAC then men, a CAC score >0 was predictive of ASCVD events beyond traditional risk factors, even among those at low risk [32,33], and was associated with a higher relative risk of mortality from ASCVD than in men [34]. It is worthy of note that, in a prospective follow-up study, a CAC score of 0 conferred a 15-year “warranty period” against mortality in individuals at low-to-intermediate risk, and better survival in those at high risk, as compared to those at low-to-intermediate risk but with any CAC score [35]. Finally, CAC was found to provide superior discrimination and risk reclassification compared with other markers [36].

More recently, the CAC score was also found to be of prognostic value in symptomatic individuals. In patients with stable chest pain (or dyspnea) from the Prospective Multicenter Imaging Study for Evaluation of Chest Pain (PROMISE) Study, CAC scoring was in fact shown to be more sensitive, but less specific than functional testing in predicting mortality and CAD events, with similar overall discriminatory ability [37]. The prognostic value for MACEs of the CAC score in symptomatic individuals was recently confirmed by a meta-analysis of 19 observational studies [38]. Among symptomatic patients from the Western Denmark Heart Registry, the ASCVD event rate increased stepwise with higher CAC scores, but regardless of whether they have obstructive or non-obstructive CAD [39]. Conversely, a negative predictive value was demonstrated for a CAC score of 0 among patients with either acute or chronic chest pain, supporting the safe avoidance of additional downstream testing [40]. However, the absence of CAC does not exclude the presence of a non-calcified plaque causing obstructive CAD and the occurrence of acute events, as shown in symptomatic patients from *The Coronary CT Angiography Evaluation for Clinical Outcomes: An International Multicenter (CONFIRM) Registry* [41]. Recent data from the Western Denmark Heart Registry showed that a sizable number of cases of obstructive CAD occurred in patients without CAC who were younger than 60 years [42].

A main limitation of CAC scoring is that it does not account for the type and pattern of calcium deposition within the vessel wall, which limits its accuracy in predicting obstructive CAD and coronary events in the individual patient [18]. This concept is supported by studies showing that a high CAC score correlates with plaque stability rather than with plaque instability [43,44,45], and that plaque stabilization upon statin treatment is associated with an increase in CAC score [46,47,48], indicating that, in patients with heavily calcified coronary arteries, the CAC score is more a marker of overall CAD burden than a predictor of a future coronary event [49]. This concept is supported by the finding that highly calcified plaques (>1000 HU) were associated with a lower risk of acute coronary syndrome (ACS) in a nested case-control study of patients with no known CAD drawn from the CONFIRM Registry [50]. Moreover, calcium density was found to be inversely related to CAD and ASCVD for a given calcification volume, which was more predictive of CVD risk when adjusted for calcium density [51]. An inverse relationship between calcium density and instability features was also observed at the individual plaque level [45].

### 2.2. Epicardial Adipose Tissue

Epicardial adipose tissue (EAT) is a unique fat depot, as it is anatomically and functionally different from other visceral and subcutaneous fat depots [52], despite sharing the embryological origin from the splanchnopleuric mesoderm with intra-abdominal fat [53]. It is located between the myocardium and the visceral pericardium, with no muscle fascia separating the fat depot and the myocardium, which share the same microcirculation [52]. These anatomical features allow fat infiltration into the myocardium and the coronary arteries, and the direct cross-talk of EAT with muscle and vessels through paracrine and vasocrine mechanisms [52]. Under physiologic conditions, EAT is protective for the myocardium through its dynamic brown fat-like function that promotes fatty acid uptake, oxidation, and thermogenesis, as well as fatty acid release, thus serving as a source of energy and heat for the myocardium and a buffer for high fatty acid levels [54]. This brown fat-like activity of EAT decreases substantially with age, with a gradual transition from thermogenesis to energy storage [52]. As with other visceral fat depots, EAT increases in obese individuals, and becomes harmful for the myocardium by acquiring a functional beige-white phenotype associated with macrophage infiltration, which results in a change in the transcriptome and secretome profile with pro-oxidant, pro-inflammatory, and pro-fibrotic effects on the heart [55]. For this reason, EAT accumulation and dysfunction is considered to be not only a marker of systemic inflammation in metabolic disorders such as obesity and type 2 diabetes, as with excess intra-abdominal fat, but also a player in the pathogenesis of CAD and other cardiac conditions such as arrhythmias and heart failure [52]. In this regard, a major role is attributed to the EAT located in close proximity to the coronary arteries, which will be discussed later.

Non-contrast CT allows for the measurement of EAT thickness and volume [56], which represents markers of visceral adiposity and ectopic fat accumulation, as they correlate with intra-abdominal as well as intra-hepatic and intra-muscular (including the myocardium) fat [52]. While echocardiography only measures EAT thickness, CT (and magnetic resonance) allows also measurement of EAT volume, which can be performed using dedicated software [52]. In addition, CT can assess EAT attenuation, a measure of EAT density expressed in HU units and ranging between −45 HU and −195 HU, which is decreased (i.e., more negative) in cases of hypertrophic and hyperplastic fat depots, and increased (i.e., less negative) in cases of fibrotic and inflamed fat depots [52].

Several studies have shown that EAT volume, as assessed by CT, is positively associated with coronary atherosclerosis. In fact, EAT volume was found to be associated with CAC in asymptomatic individuals from the Early Identification of Subclinical Atherosclerosis using Non-invasivE Imaging Research (EISNER) Trial [57], whereas previous reports from the population-based Rotterdam Study [58] and the influence of EPICardial adipose tissue in HEART disease (EPICHEART) Study [59] showed that this relationship was only significant in men. Moreover, the EAT volume was shown to be associated with CAC progression, independent of measures of adiposity, in patients with [60] and without [61] diabetes. However, in the HNR Study, the association of EAT volume with CAC progression was found to be stronger in younger individuals with lower CAC scores at baseline [62], suggesting that EAT expansion is a predictor of early atherosclerosis. Indeed, the EAT volume was shown to be larger in the presence of mixed or non-calcified plaques than with calcified plaques (or no plaques) [63], and to correlate with plaque instability features independent of measures of adiposity [64]. In addition, EAT volume was found to correlate with obstructive CAD independently of CAC score in both asymptomatic [65] and symptomatic [62,66,67] individuals undergoing CCTA, and even in patients with a CAC score = 0 [68]. Finally, a meta-analysis of 70 studies comprising 41,534 subjects, mainly derived from community-based or hospital-based populations with low-to-intermediate pretest CAD probability, showed that EAT volume was independently associated with obstructive CAD (coronary stenosis and myocardial ischemia) and CAD events, whereas the correlation with CAC was only borderline significant [69].

Altogether, these findings indicate that EAT quantification by non-contrast CT adds to the prognostic value of CAC scoring and, therefore, it may be routinely performed to better estimate the risk of obstructive CAD and CAD events.

## 3. Contrast-Enhanced CT

During the last decade, coronary CT angiography (CCTA) has emerged as a useful tool in CAD detection by allowing for the non-invasive assessment of the presence and extent of coronary artery stenosis, eventually combined with the evaluation of its functional significance through the measurement of FFR derived from CT (FFR_CT_), or CT perfusion imaging (CTPI). Moreover, and possibly more importantly, by also imaging the vessel wall, CCTA has been found to provide information on the biological processes driving coronary atherosclerosis which are not fully reflected by the severity of lumen narrowing and/or myocardial ischemia, and allow a more accurate diagnostic and prognostic assessment. It is in fact known that a significant proportion of acute CAD events result from originally non-obstructive, unstable plaques which subsequently progress and undergo fibrous cap rupture and thrombus formation with consequent lumen occlusion [70,71]. This points to the importance of assessing the plaque burden, composition, and features of instability, as well as changes in perivascular adipose tissue (PVAT), indicating an active process of vascular inflammation.

### 3.1. Lumen Stenosis

Coronary stenosis is the hallmark of CAD, as it is associated with reduced coronary blood flow and myocardial ischemia at a threshold of ~50% and ~80%, respectively [72]. Conventionally, a stenosis of 70% or more is considered to be hemodynamically significant and worthy of therapeutic intervention with invasive procedures and, hence, assessing the extent of lumen narrowing is of pivotal importance for evaluating CAD severity [73].

This is the reason why ICA is the gold standard for detecting obstructive CAD, and why CCTA has been proposed as a safe, non-invasive gatekeeper for identifying patients who warrant subsequent ICA, as an alternative to functional testing [73]. In fact, CCTA provides a three-dimensional imaging of the arterial lumen that allows for the quantification of stenosis and the classification of patients according to the degree of maximal stenosis based on the CAD Reporting and Data System (CAD-RADS) (Table 1) [74]. A meta-analysis including small-size single-center studies first showed that the diagnostic performance of CCTA was approximately similar to that of ICA [75]. This was subsequently confirmed by larger single-center or multi-center studies in patients referred for ICA, which demonstrated that CCTA was effective in detecting, and especially ruling out, obstructive CAD [76,77,78,79], although it was found to somewhat overestimate its severity [79]. In addition, a number of studies comparing CCTA with ICA as an initial imaging approach in patients with suspected CAD showed that CCTA resulted in less invasive procedures and a higher diagnostic yield than ICA, with similar clinical outcomes in terms of major adverse cardiovascular events (MACEs) [80,81,82]. Finally, CCTA was found to have the potential for guiding the decision-making between percutaneous coronary intervention (PCI) and coronary artery by-pass grafting in patients with complex CAD [83]. In particular, CCTA was shown to be as accurate as ICA in the assessment of CAD anatomical complexity for calculating the SYNergy between percutaneous coronary intervention with TAXus and cardiac surgery (SYNTAX) scores [84,85], or the SYNTAX-II score [86], which integrates anatomical and clinical features [87].

As compared with functional tests, CCTA was shown to have a higher specificity and sensitivity with ICA >50% diameter stenosis as the reference standard [88,89], and the highest sensitivity but the lowest specificity with invasive FFR <0.80 as the reference standard [90]. In low- and/or intermediate-risk individuals with acute chest pain and normal ECG and troponin values, CCTA resulted in similar outcomes and resource use as functional testing, as shown in the American College of Radiology Imaging Network-Pennsylvania (ACRIN-PA) Multicenter Trial [91], the Prospective Randomized Outcome trial comparing radionuclide Stress myocardial Perfusion imaging, the ECG-gated coronary CT angiography (PROSPECT) Study [92], and the Prospective First Evaluation in Chest Pain (PERFECT) Trial [93]. However, improved outcomes with CCTA compared with functional tests were reported in the Rule Out Myocardial Infarction/Ischemia Using Computer Assisted Tomography (ROMICAT)-II Study [94] and the CArdiac cT in the treatment of acute CHest pain (CATCH) Trial [95], whereas the CT Coronary Angiography Compared to Exercise ECG (CT-COMPARE) Study showed a better performance with lower costs with CCTA [96]. Studies in low- and/or intermediate-risk individuals with stable chest pain also showed similar, if not better, outcomes with CCTA compared with functional tests. No difference was found in the PROMISE Study [97], and in an earlier small-size study comparing CCTA and MPI with single-proton emission computed tomography (PET) scanning [98]. Conversely, the superiority of CCTA in terms of CAD morbidity and mortality over a 4.8-year follow-up was observed in the Scottish Computed Tomography of the Heart (SCOT-HEART) Trial, which was associated with no increase in the rate of ICA or coronary revascularization [99], whereas less symptoms with CCTA than with functional tests were reported in the Computed Tomography vs. Exercise Testing in Suspected Coronary Artery Disease (CRESCENT) [100] and the Cardiac CT for the Assessment of Pain and Plaque (CAPP) [101]. A meta-analysis including most of the above studies in patients with either acute or stable chest pain showed that anatomical testing with CCTA as the initial non-invasive diagnostic modality resulted in a lower risk of non-fatal myocardial infarction, but not MACEs or all-cause mortality, as compared with the usual care with functional testing at the expense of a more frequent use of invasive procedures [102]. Moreover, a systematic review in patients with acute or stable chest pain showed that CCTA is cost-effective when compared with the standard of care, including functional testing [103].

Several studies have shown the prognostic value of CCTA in terms of the prediction of CAD events [104,105,106,107,108,109], which was found to be higher in the PROMISE Study using the CAD-RADS compared to traditional stenosis categories, with CAD-RADS also adding an incremental value beyond ASCVD risk score and CAC score [110]. The powerful predictive capacity of CCTA is due to its ability to identify both non-obstructive and obstructive CAD. In fact, a subsequent analysis of the PROMISE Study showed that CCTA had a higher discriminatory ability to predict CAD events than functional testing because of its capacity to detect non-obstructive lesions (see below) [111]. However, a meta-analysis of 21 studies in patients with suspected or known CAD showed a similar prognostic value for fatal and non-fatal myocardial infarctions between CCTA and stress nuclear MPI [112]. Moreover, in patients with acute chest pain from the ROMICAT Study, early triage with CCTA was effective in identifying the large proportion of individuals without CAD (50%), who had no ACS and no need for invasive anatomic testing [113]. A large meta-analysis showed a similar negative prognostic value of CCTA and functional tests after adjusting for population event risk [114], but a normal CCTA was found to be associated with an excellent prognosis over a follow-up of 5 [104,105,107,115] and even 10 [116] years.

### 3.2. Myocardial Ischemia

The extent of myocardial ischemia is certainly dependent on the severity of lumen stenosis. However, the 70% threshold for hemodynamically significant coronary stenosis does not necessarily imply the presence of myocardial ischemia which requires invasive therapeutic interventions, thus suggesting the need for assessing the functional significance of lumen narrowing [73]. The accuracy of ICA to indicate the need for coronary revascularization is greatly increased by combining it with invasive FFR assessment, which allows for the more precise assessment of the hemodynamic consequences of the coronary stenoses compared to non-invasive functional tests [117]. In fact, ischemia assessed by functional testing was not associated with outcomes after adjusting for CAD severity in the International Study of Comparative Health Effectiveness with Medical and Invasive Approaches (ISCHEMIA) Trial [118]. Conversely, the use of invasive FFR to guide coronary revascularization resulted in improved clinical outcomes compared with ICA in the Percutaneous Coronary Intervention of Functionally Non-significant Stenosis (DEFER) Trial [119] and the Fractional Flow Reserve Versus Angiography for Multivessel Evaluation (FAME) Study [120].

Coupling CCTA with either FFR_CT_ or CTPI represents a suitable non-invasive alternative to invasive FFR. While computational flow dynamic or machine learning techniques are applied to derive FFR_CT_, static or dynamic imaging acquisitions under rest and stress conditions (or vice versa) are required for CTPI [121]. Several meta-analyses have provided evidence that both FFR_CT_ [90,122,123,124] and CTPI [122,123,125] are valuable tools for detecting hemodynamically significant coronary stenosis compared with invasive FFR. In particular, two of these meta-analyses reported similar sensitivity and specificity for FFR_CT_ and/or CTPI compared to other functional imaging modalities [122,125], whereas the others showed that FFR_CT_ and/or CTPI improved the diagnostic accuracy of CCTA by increasing the specificity [90,123,124].

For FFR_CT_, this was confirmed in the large cohort of the Assessing Diagnostic Value of Non-invasive FFR_CT_ in Coronary Care (ADVANCE) registry [126], and in participants in the NXT [127] and Prospective Comparison of Cardiac PET/CT, SPECT/CT Perfusion Imaging and CT Coronary Angiography with Invasive Coronary Angiography (PACIFIC) [128] trials. In addition, the 90-day [126] and 1-year [129] outcome data from the ADVANCE registry showed that FFR_CT_ significantly modified patients’ management with the safe deferral of invasive evaluation in those with negative values (i.e., >0.80). Similarly, in the NXT Trial, FFR_CT_ significantly ameliorated the ability of CCTA to predict long-term outcomes driven by planned and unplanned revascularization [130]. Moreover, the FFR_CT_ Planner is a novel tool that allows for the virtual stenting of coronary stenoses and the prediction of post-PCI FFR [131]. This might be useful in patient selection and procedural planning because of the important prognostic implications of post-PCI FFR [132], which remains suboptimal in a substantial proportion of individuals [133]. Post-PCI FFR was also shown to correlate with vessel/lesion-specific myocardial mass, in addition to the coronary volume to mass ratio [134], which can be quantified on CCTA using dedicated algorithms [135]. However, costs increase substantially when using FFR_CT_, which should be reserved for patients with an intermediate-to-high pre-test probability of CAD with significant or uncertain stenosis at CCTA, who showed the highest post-test probability in a recent meta-analysis [136].

For CPTI, both static and dynamic procedures were shown to provide incremental value over CCTA for the detection of hemodynamically significant CAD [137,138,139,140,141,142,143]. Though no study was performed comparing static and dynamic CPTI head-to-head, meta-analyses seem to indicate a somewhat higher accuracy for the latter [144,145]. In the CRESCENT-II, dynamic CTPI was found to be superior to functional tests in patients with suspected CAD [146]. Moreover, dynamic CTPI allows the quantification of myocardial blood flow and stress myocardial blood flow ratio, which provided further incremental value over CCTA for the diagnosis and stratification of patients [147,148].

Head-to-head comparisons of FFR_CT_ and CTPI showed that these two procedures have a similar performance [149,150,151]. However, FFR_CT_ offers practical advantages over CTPI, which represents a suitable alternative when FFR_CT_ is not available or technically not possible because of insufficient CCTA image quality or prior revascularization.

### 3.3. Plaque Burden, Composition and Instability Features

Lumen narrowing is due to the progressive growth of the atheroma, which initially increases in size by expanding outward as a compensatory mechanism to preserve luminal integrity and maintain coronary blood flow, i.e., the so-called Glagov phenomenon [152]. Therefore, significant atherosclerotic lesions may even be present with no or non-obstructive coronary stenosis. Moreover, non-obstructive, not hemodynamically significant lesions cause acute CAD events to a similar, if not higher, extent compared with obstructive lesions with clearly reduced coronary blood flow [70,71]. This is due to the much higher prevalence of non-obstructive plaques, a few of which rapidly progress by increasing in volume [153], and undergo fibrous cap rupture that exposes the necrotic core to the bloodstream, resulting in thromboembolism [154]. These “unstable” plaques differ from those experiencing no or slow progression (“stable” plaques) in terms of both composition and the presence of instability features. Based on their composition, atherosclerotic plaques are usually classified as lipid, fibro-fatty, fibrous, and fibro-calcific. As the higher the lipid content the higher is the risk of progression and rupture, lipid and fibro-fatty plaques are the most common and unstable, whereas the fibrous and fibro-calcific plaques are the most stable [155]. Moreover, selected features have been shown to characterize high-risk plaques, including (a) positive remodeling, defined as a ratio of the vessel area at the site of plaque compared with the area at a normal reference site >1, i.e., the Glagov phenomenon of outward plaque expansion [156]; (b) thin-cap fibroadenoma (TCFA), defined as a large necrotic core with a fibrous cap <65 mm, i.e., the rupture-prone lesion [157]; (c) spotty calcification, defined as calcium deposits with a size of <3 mm or an arc of <90°, i.e., the calcification pattern associated with plaque inflammation [158]; and (d) intraplaque vasa vasorum [157]. These findings indicate the need for imaging the vessel wall to detect and possibly quantify plaques, to assess plaque composition, and to identify the presence of instability features that may predict plaque progression and rupture. As previously stated, ICA is not suitable for assessing and characterizing changes occurring in the vessel wall and plaques enlarging in an outward direction may be invisible to this procedure. However, there are several methods that are currently available for plaque imaging, including intravascular ultrasonography (IVUS), IVUS with virtual histology (IVUS-VH), elastography, near-infrared spectroscopy (NIRS), and optical coherence tomography (OCT). All of these technologies are able to assess coronary plaque burden and composition, and to identify plaque instability features, with fibrous cap thickness for detecting TCFA being ensured only by OCT, which, however, is limited by the lowest tissue penetration [159].

**Figure 1:**
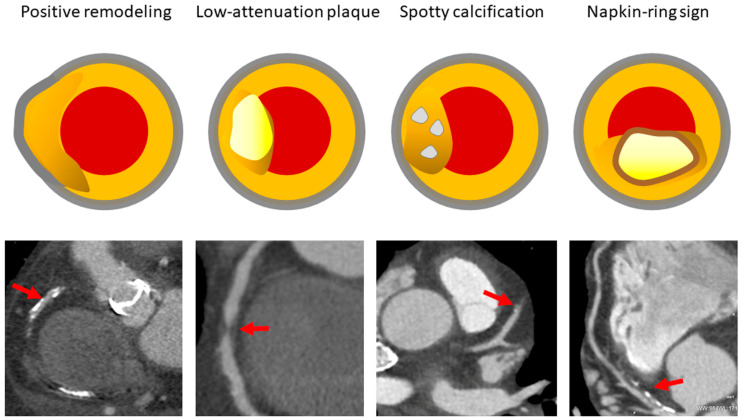
Illustrations and representative CCTA images of plaque instability features. CCTA = coronary computed tomography angiography. The arrows indicate the corresponding plaque instability feature, i.e., positive remodeling, low-attenuatiuon plaque, spotty calcification and napkin-ring sign.

The invasive nature of these methods limits their routine use and makes CCTA an attractive non-invasive alternative for plaque characterization, as it provides three-dimensional images of the vessel wall for the assessment of plaque volume, composition and instability features. A semiquantitative CCTA imaging analysis using dedicated software allows for the measurement of total plaque volume, percent atheroma volume, and total atheroma volume normalized for vessel length [160,161]. Of these measures, percent atheroma volume was shown to be less affected by body surface area than the other two, suggesting that it may be the preferred method for reporting the coronary atherosclerotic burden [162]. Moreover, based upon density/attenuation values adapted to lumen contrast intensity, plaques can be classified into calcified (HU ≥ 150, usually >400 HU) and non-calcified (HU < 150), the latter including fibrous (60–149 HU), lipid or low-attenuation (<60 HU), and fibro-fatty [163], as validated against histology [164]. Finally, several plaque instability features can be detected by CCTA [165], including positive remodeling, low-attenuation plaque, spotty calcification, and a napkin-ring sign (Figure 1), the last of which is defined as a low attenuation region surrounded by a higher-attenuation ring, which is believed to correspond to a necrotic core surrounded by a fibrous cap [166,167]. Several studies have shown a very high sensitivity and specificity of CCTA compared with IVUS [168,169,170,171,172,173], as confirmed by two meta-analyses [174,175]. A good correlation for plaque geometry and composition was also reported with VH-IVUS [176,177,178], although CCTA tended to overestimate lumen, vessel, and both calcified and noncalcified plaques [176] and could not identify TCFA due to limitations in the spatial resolution [177].

All of these plaque characteristics were shown to predict outcomes independent of the presence of obstructive CAD. A meta-analysis of 11 studies showed that the overall plaque burden, as assessed by CCTA, is associated with MACEs [179]. Moreover, plaque burden quantified by CCTA correlated with ischemia detected by invasive FFR [180] and CTPI [181], and both total plaque volume [182,183] and percent atheroma volume [162,184] predicted outcomes. Total plaque score [185] and total plaque volume [115] had an incremental value above CAD severity. Regarding plaque type, data from the CONFIRM Registry indicated that the number of proximal segments with either calcified or mixed plaques and >50% stenosis predicted mortality beyond conventional clinical risk models [106], whereas other CCTA studies reported that non-calcified and mixed plaques had a higher prognostic value than from plaques [186,187,188]. Plaque instability features were also shown to correlate with invasively measured FFR [189,190] and to predict outcomes, including positive remodeling [191,192,193,194], low attenuation plaque [191,192,193,194], spotty calcification [182], and a napkin-ring sign [193,195]. In the ROMICAT-II, the presence of high-risk plaques (HRPs) predicted ACS independent of significant CAD and clinical risk factors [196]. However, in the SCOT-HEART Trial, these adverse plaque features were not a predictor of coronary evens independent of CAC score [194]. Moreover, the incremental information provided by HRP characteristics appears to be modest in individual patients. In fact, in both the PROMISE Study [193] and the SCOT-HEART Trial [194], only a few patients with HRP had adverse events, which also occurred in those without HRPs, and the risk was 6.4% vs. 2.4% and 4.1% vs. 1.4%, respectively, in the presence vs. absence of HRPs. This was confirmed by studies with VH-IVUS in participants in the PROSPECT Study [71] and the PROSPECT-II Study [197], in which VH-IVUS was combined with NIRS.

### 3.4. Perivascular Adipose Tissue

The EAT surrounding the coronary arteries, called PVAT, differs from the rest of the EAT both morphologically and functionally [198]. It is embedded in the vascular wall, being contiguous with the adventitial layer in large vessels or an integral part of the vascular wall itself in smaller vessels [199]. Due to these anatomic characteristics, PVAT participates in a bidirectional interplay with the vascular wall, with outside to inside signals involved in the regulation of vascular tone, vascular smooth muscle cell (VSMC) migration, endothelial cell activation, and oxidative stress and inflammation, and inside to outside signals that are involved in the regulation of adipocyte growth, differentiation, and lipid accumulation [200]. The PVAT acts as a sensor of signals form the vascular wall, which drive changes in the surrounding fat depot. Under normal conditions, inside to outside signals promote adipogenesis and lipogenesis and trigger adiponectin release, which in turn exerts a vasoprotective role. In contrast, inflamed vessels secrete cytokines that suppress adipogenesis and stimulate lipolysis in the PVAT, with a consequent reduction in adipocyte size and an increased intracellular and extracellular water content, the latter of which is due to enhanced microvascular permeability. These phenotypic changes result in a pro-inflammatory shift of PVAT that in turn amplifies vascular inflammation and injury [200]. Thus, at variance with changes in the rest of the EAT, which reflect obesity-related systemic inflammation, PVAT changes represent a marker of local inflammation, which is the key biological process driving the plaque progression and the risk of events [201].

As a consequence, imaging PVAT may provide important information for further classifying patients beyond CAD extent and severity and plaque burden and instability features. The gold standard in in vivo imaging of tissue inflammation is measuring fluorodeoxyglucose (FDG) or ^18^F-sodium fluoride (^18^F-NaF) uptake using PET, which has been shown to be increased in patients with significant coronary stenosis [202], and in those with ruptured plaques [203], respectively. The shift in PVAT composition from the lipid to the aqueous phase triggered by signals originated in the inflamed vessel results in increased tissue attenuation that can be detected prospectively or retrospectively using CCTA [200], with values that were shown to correlate with ^18^F-NaF PET uptake in stable patients with HRPs [203]. Using a radiotranscriptomic approach, the presence of large adipocytes was associated with a PVAT attenuation in the more negative range (towards −190 HU), whereas the presence of small adipocytes correlated with a PVAT attenuation in the less negative range (towards −30 HU) [198]. the attenuation of PVAT, as measured by CCTA, was shown to correlate with total volumes and the burden of non-calcified plaque in individuals with stable chest pain [204], and to be higher around culprit lesions compared with non-culprit lesions of patients with ACS and the lesions of matched controls with stable CAD [205]. Importantly, the increased PVAT attenuation associated with coronary inflammation is dynamic, as it was shown to normalize after PCI or initiation of statin treatment [200]. However, crude measures of PVAT attenuation require corrections for several anatomical, clinical and technical factors. An artificial intelligence-based image analysis of three-dimensional changes of PVAT attenuation allowed the obtaining of a measure of weighted attenuation shifts called the fat attenuation index (FAI). For this purpose, the PVAT space was defined as the EAT located within a radial distance from the outer vessel wall equal to the diameter of the adjacent coronary vessel [198]. The measurement of FAI was originally limited to the proximal 40 mm segments of the three main coronary arteries, where it was shown to be significantly higher in patients with than in those without CAD, independent of the CAC score [198]. It was subsequently applied to any coronary segment, and particularly around individual plaques [200]. A new medical device, the CaRi-Heart^®^, integrates standardized FAI mapping together with clinical risk factors and plaque metrics to provide individualized ASCVD risk prediction. This tool was tested in a US population and then validated in a European population for its ability to improve risk discrimination over a clinical risk factor-based model [206]. In a post-hoc analysis of the Cardiovascular RISk Prediction using Computed Tomography (CRISP-CT) Study, a PVAT FAI greater than the calculated cut-off of −70.1 HU was associated with an increased risk of all-cause and cardiovascular mortality in both a German derivation cohort and a US validation cohort, and improved risk discrimination above clinical risk factors and the CCTA-derived CAD extent and HRP features [207]. In another study, a post-hoc analysis of the CRISP-CT Study found that a FAI greater than −70.1 HU predicted adverse outcomes not only in patients with at least one HRP feature, thus identifying a small group at very high risk, but also in the large group of those without any HRP feature [208]. Finally, extending the radiotranscriptomic approach to derive new signatures that reflect PVAT structural remodeling beyond inflammation, a radiomic profiling was developed and validated in patient cohorts from three different studies for the improvement of MACE prediction above clinical risk factors, CAC score, coronary stenosis, and HRP features [209].

Taken together, these findings indicate that FAI, especially if combined with an HRP feature, represents a powerful tool to improve risk stratification and treatment allocation in patients undergoing CCTA. By detecting coronary inflammation, it has the potential to identify those individuals with a residual risk for future CAD events among those at apparently low risk, thus resulting in the long-term reduction of mortality and morbidity from ASCVD [210].

## 4. Cardiac CT Positioning in Current Guidelines

Both non-contrast CT for measuring CAC and CCTA for assessing CAD extent and severity, plaque composition, HRP features, and eventually FFR_CT_, are included in the diagnostic and prognostic flow chart under the current guidelines.

### 4.1. CAC Score

In 2010, the American College of Cardiology (ACC)/American Heart Association (AHA) guidelines recommended (for the first time) the inclusion of the CAC measurement by CT in ASCVD risk assessments in asymptomatic adults at low-to-intermediate (6–10%) or intermediate (10–20%) 10-year risk [211]. The 2019 ACC/AHA Guideline on the Primary Prevention of Cardiovascular Disease confirms the role of CAC in the risk stratification of asymptomatic adults [212]. In fact, in the decision flowchart for initiating statin treatment, these guidelines recommend considering the measuring of CAC in borderline (5–7.5%) 10-year risk individuals if risk enhancers are present and in intermediate (7.5–20%) 10-year risk individuals if the decision is uncertain based on risk enhancers. Reclassification to the high-risk category after CAC measurement may also guide anti-hypertensive treatment and aspirin use. The 2021 European Society of Cardiology (ESC) guidelines on Cardiovascular Disease Prevention also recommend the consideration of CAC scoring to improve the risk classification around treatment decision thresholds [213]. The measurement of CAC is especially indicated in asymptomatic individuals at moderate risk of suffering from diabetes [214], a condition characterized by a higher calcification burden [215].

More recently, CAC measurement has also been included in the diagnostic flowchart for symptomatic individuals presenting with chest pain. The 2021 ACC/AHA guidelines for the evaluation and diagnosis of chest pain recommend the use of the CAC score as a risk modifier in patients with stable chest pain at low-to-intermediate risk [216]. In particular, in these individuals, CAC scanning can provide further information, in addition to age, sex, and symptoms, for estimating the pre-test probability of obstructive CAD, and indicate the need of performing functional and/or anatomic testing, including CCTA, which can be avoided in the case of a CAC score = 0. The same recommendation regarding the use of CAC score is contained in the 2019 ESC Guidelines for the Diagnosis and Management of Chronic Coronary Syndromes, although it is noted that the CAC score is a weak predictor of obstructive CAD in the individual patient [217].

### 4.2. CCTA

The 2021 ACC/AHA guidelines for the evaluation and diagnosis of chest pain endorse the use of CCTA in patients with both acute and stable chest pain, according to the pre-test probability of CAD [216]. Specifically, in intermediate-risk patients with acute chest pain and no known CAD after a negative or inconclusive stress test, CCTA is useful for the exclusion of atherosclerotic plaque and obstructive CAD, whereas its use is reasonable in those with previous mildly abnormal stress test results. In intermediate-risk patients with acute chest pain and known nonobstructive CAD, CCTA can be useful to determine the progression of atherosclerotic plaque and obstructive CAD, whereas its use is indicated in those with prior coronary artery by-pass surgery to evaluate for graft stenosis or occlusion. In intermediate-to-high-risk patients with stable chest pain and no known CAD, CCTA is recommended for CAD diagnosis, risk stratification, and guiding treatment decisions, whereas it is reasonable after an inconclusive or negative (with high clinical suspicion) functional testing, and useful in addition to a stress test. In patients with stable chest pain and known obstructive CAD, CCTA is reasonable to evaluate bypass grafts or stent patency in those with previous coronary revascularization and the progression of CAD in those with new or recurrent chest pain. Moreover, FFR_CT_ is useful in guiding decision-making for coronary revascularization in intermediate-risk patients with acute or stable chest pain and no known CAD, with a coronary artery stenosis of 40% to 90% in a proximal or middle coronary artery on CCTA.

The 2019 ESC guidelines for the diagnosis and management of chronic coronary syndromes recommend CCTA or non-invasive functional imaging as the initial test for diagnosing CAD in symptomatic patients in whom obstructive CAD cannot be excluded by clinical assessment alone, based on the clinical likelihood of CAD and other patient characteristics that influence test performance, local expertise, and the availability of tests [207,218].

The 2021 Expert Consensus Document on CCTA in stable chest pain of the Society of Cardiovascular Computed Tomography (SCCT) [219] confirms the appropriateness to perform CTA as the first line test for evaluating patients with unknown or known CAD who present with stable typical or atypical chest pain, or possible anginal equivalent, after an inconclusive functional test and post-revascularization procedures, as well as to perform FFR_CT_ or CTPI to evaluate the functional significance of intermediate stenoses on CCTA, particularly in the setting of multivessel disease, to help guide ICA referral and revascularization treatment planning. In addition, this document considers it to be appropriate to perform CCTA in selected asymptomatic high-risk individuals, especially in those who have a higher likelihood of having a large amount of noncalcified plaque, prior non-coronary cardiac or non-cardiac surgery, and in diagnosing other cardiac disease conditions.

The 2022 SCCT 2022 expert consensus document on the use of CCTA for patients presenting to the emergency department with acute chest pain recommend its use in CCTA in patients at low-to-intermediate risk for CAD (and in selected patients at high risk or very low risk), and to classify based on the CAD-RADS and the presence of HRPs features to make a decision on whether to discharge, observe, or to proceed to further testing (functional or FFR_CT_) or IVA [220]. Another SCCT expert consensus document supports the use of CCTA and eventually FFR_CT_ and CTPI for pre-procedural planning of coronary revascularization [221].

## 5. Conclusions

**Figure 2:**
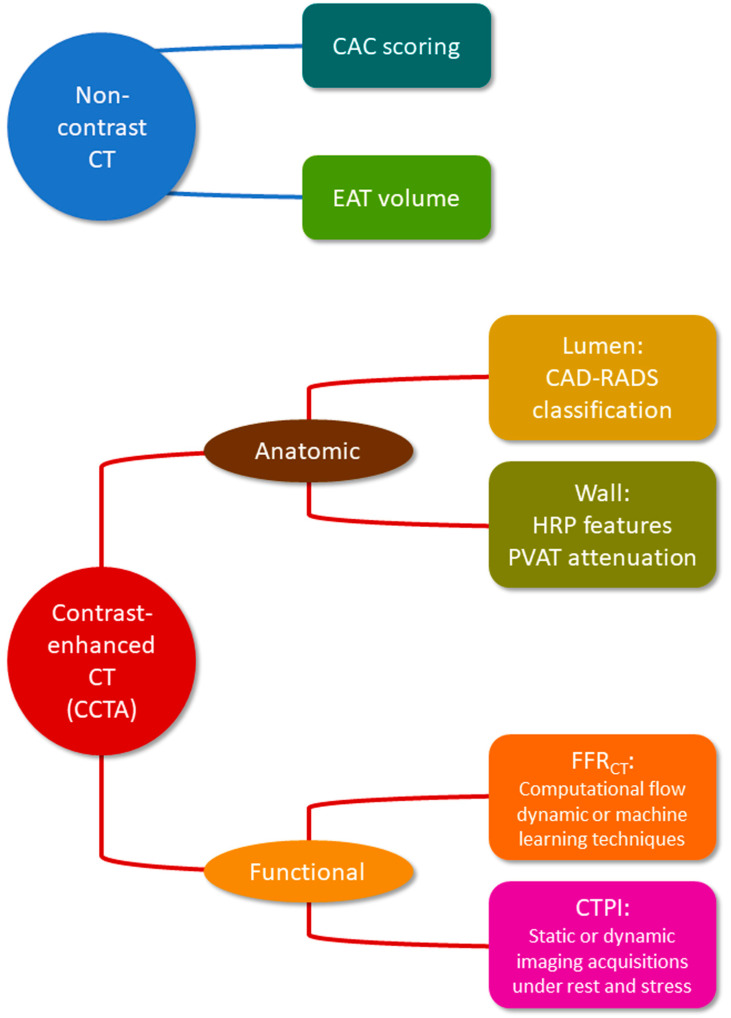
Role of cardiac CT imaging in CAD evaluation. CT = computed tomography; CCTA = coronary computed tomography angiography; CAC = coronary artery calcium; EAT = epicardial adipose tissue; CAD-RADS = Coronary Artery Disease Reporting and Data System; HRP = high-risk plaque; PVAT = perivascular adipose tissue; FFR_CT_ = computed tomography-derived fractional flow reserve; CTPI = computed tomography perfusion imaging.

Cardiac CT imaging has emerged as a powerful non-invasive tool for risk stratification, as well as the detection and characterization of CAD (Figure 2).

The increasing use of cardiac CT has been favored by the innovations in scanner technology and acquisition protocols that have resulted in decreased radiation dose and increased spatial and temporal resolution, as well as by the development of dedicated software that have allowed the reproducible quantitation of plaque features and fat metrics.

In particular, CAC scoring, as assessed by non-contrast CT, is considered to be the best marker of subclinical atherosclerosis, and its use is recommended for the refinement of risk assessment in individuals at low-to-intermediate risk, both asymptomatic, to inform lipid-lowering, anti-hypertensive and anti-platelet treatment, and symptomatic, to indicate the need to perform functional and/or anatomic testing. The concurrent assessment of EAT volume may provide additional prognostic information by reflecting systemic inflammation.

Moreover, CCTA has become a gate-keeper to ICA and coronary revascularization in patients with acute chest pain and normal ECG and troponin values, as well as an accurate method for diagnostic and prognostic purposes in those with stable chest pain. In fact, CCTA allows for the assessment of not only the extent of lumen stenosis, but also its hemodynamic significance if combined with FFR_CT_ or CTPI, thus guiding decision-making for coronary revascularization. Even more importantly, CCTA provides a unique incremental value over functional testing and ICA by imaging the vessel wall, thus allowing for the assessment of (a) plaque burden, composition, and instability features with a very high sensitivity and specificity compared with invasive intravascular methods; and (b) PVAT attenuation, which is a reliable and dynamic marker of vascular inflammation, i.e., the key biological process underlying coronary atherosclerosis. Combining all of these measures has the potential to identify, among the non-obstructive lesions that are responsible for a significant proportion of acute CAD events, those at a high risk of progression to plaque rupture.

## Figures and Tables

**Table 1:** CAD-RADS classification for patients with acute and stable chest pain (modified from [74]).

CAD-RADS Category	Degree of Maximal Coronary Stenosis (%)	Interpretation in Acute Chest Pain (ACS)	Interpretation in Stable Chest Pain (CAD)
**0**	0	Highly unlikely	Absence of CAD
**1**	1–24	Highly unlikely	Minimal non-obstructive CAD
**2**	25–49	Unlikely	Mild non-obstructive CAD
**3**	50–69	Possible	Moderate stenosis
**4A**	One or two vessels: 70–99	Likely	Severe stenosis
**4B**	Left main artery: >50 or three vessels ≥70	Likely	Severe stenosis
**5**	100	Very likely	Total occlusion
**N**	Non-diagnostic	Cannot be excluded	Cannot be excluded

CAD-RADS = Coronary Artery Disease Reporting and Data System; ACS = acute coronary syndrome; CAD = coronary artery disease.

## Data Availability

Not applicable.

## References

[1] Tsao C.W., Aday A.W., Almarzooq Z.I., Alonso A., Beaton A.Z., Bittencourt M.S., Boehme A.K., Buxton A.E., Carson A.P., Commodore-Mensah Y. (2022). Heart Disease and Stroke Statistics-2022 Update: A Report from the American Heart Association. Circulation.

[2] Gao Y., Isakadze N., Duffy E., Sheng Q., Ding J., MacFarlane Z.T., Sang Y., McClure S.T., Selvin E., Matsushita K. (2022). Secular Trends in Risk Profiles Among Adults with Cardiovascular Disease in the United States. J. Am. Coll. Cardiol..

[3] Sofogianni A., Stalikas N., Antza C., Tziomalos K. (2022). Cardiovascular Risk Prediction Models and Scores in the Era of Personalized Medicine. J. Pers. Med..

[4] Lloyd-Jones D.M. (2010). Cardiovascular risk prediction: Basic concepts, current status, and future directions. Circulation.

[5] Devon H.A., Rosenfeld A., Steffen A.D., Daya M. (2014). Sensitivity, specificity, and sex differences in symptoms reported on the 13-item acute coronary syndrome checklist. J. Am. Heart Assoc..

[6] Canto J.G., Shlipak M.G., Rogers W.J., Malmgren J.A., Frederick P.D., Lambrew C.T., Ornato J.P., Barron H.V., Kiefe C.I. (2000). Prevalence, clinical characteristics, and mortality among patients with myocardial infarction presenting without chest pain. JAMA.

[7] Scirica B.M. (2013). Prevalence, incidence, and implications of silent myocardial infarctions in patients with diabetes mellitus. Circulation.

[8] Arbab-Zadeh A. (2012). Stress testing and non-invasive coronary angiography in patients with suspected coronary artery disease: Time for a new paradigm. Heart Int..

[9] Mintz G.S., Guagliumi G. (2017). Intravascular imaging in coronary artery disease. Lancet.

[10] Desai N.R., Bradley S.M., Parzynski C.S., Nallamothu B.K., Chan P.S., Spertus J.A., Patel M.R., Ader J., Soufer A., Krumholz H.M. (2015). Appropriate Use Criteria for Coronary Revascularization and Trends in Utilization, Patient Selection, and Appropriateness of Percutaneous Coronary Intervention. JAMA.

[11] Patel M.R., Peterson E.D., Dai D., Brennan J.M., Redberg R.F., Anderson H.V., Brindis R.G., Douglas P.S. (2010). Low diagnostic yield of elective coronary angiography. N. Engl. J. Med..

[12] Stocker T.J., Deseive S., Leipsic J., Hadamitzky M., Chen M.Y., Rubinshtein R., Heckner M., Bax J.J., Fang X.M., Grove E.L. (2018). Reduction in radiation exposure in cardiovascular computed tomography imaging: Results from the PROspective multicenter registry on radiaTion dose Estimates of cardiac CT angIOgraphy iN daily practice in 2017 (PROTECTION VI). Eur. Heart J..

[13] Wong N.D., Budoff M.J., Ferdinand K., Graham I.M., Michos E.D., Reddy T., Shapiro M.D., Toth P.P. (2022). Atherosclerotic cardiovascular disease risk assessment: An American Society for Preventive Cardiology clinical practice statement. Am. J. Prev. Cardiol..

[14] Demer L.L., Tintut Y. (2008). Vascular calcification: Pathobiology of a multifaceted disease. Circulation.

[15] Mori H., Torii S., Kutyna M., Sakamoto A., Finn A.V., Virmani R. (2018). Coronary Artery Calcification and its Progression: What Does it Really Mean?. JACC Cardiovasc. Imaging.

[16] Aikawa E., Nahrendorf M., Figueiredo J.L., Swirski F.K., Shtatland T., Kohler R.H., Jaffer F.A., Aikawa M., Weissleder R. (2007). Osteogenesis associates with inflammation in early-stage atherosclerosis evaluated by molecular imaging in vivo. Circulation.

[17] Shanahan C.M. (2007). Inflammation ushers in calcification: A cycle of damage and protection?. Circulation.

[18] Pugliese G., Iacobini C., Blasetti Fantauzzi C., Menini S. (2015). The dark and bright side of atherosclerotic calcification. Atherosclerosis.

[19] Otsuka F., Sakakura K., Yahagi K., Joner M., Virmani R. (2014). Has our understanding of calcification in human coronary atherosclerosis progressed?. Arterioscler. Thromb. Vasc. Biol..

[20] Agatston A.S., Janowitz W.R., Hildner F.J., Zusmer N.R., Viamonte M., Detrano R. (1990). Quantification of coronary artery calcium using ultrafast computed tomography. J. Am. Coll. Cardiol..

[21] Greenland P., Blaha M.J., Budoff M.J., Erbel R., Watson K.E. (2018). Coronary Calcium Score and Cardiovascular Risk. J. Am. Coll. Cardiol..

[22] Bild D.E., Detrano R., Peterson D., Guerci A., Liu K., Shahar E., Ouyang P., Jackson S., Saad M.F. (2005). Ethnic differences in coronary calcification: The Multi-Ethnic Study of Atherosclerosis (MESA). Circulation.

[23] Detrano R., Guerci A.D., Carr J.J., Bild D.E., Burke G., Folsom A.R., Liu K., Shea S., Szklo M., Bluemke D.A. (2008). Coronary calcium as a predictor of coronary events in four racial or ethnic groups. N. Engl. J. Med..

[24] Loria C.M., Liu K., Lewis C.E., Hulley S.B., Sidney S., Schreiner P.J., Williams O.D., Bild D.E., Detrano R. (2007). Early adult risk factor levels and subsequent coronary artery calcification: The CARDIA Study. J. Am. Coll. Cardiol..

[25] Carr J.J., Jacobs D.R., Terry J.G., Shay C.M., Sidney S., Liu K., Schreiner P.J., Lewis C.E., Shikany J.M., Reis J.P. (2017). Association of Coronary Artery Calcium in Adults Aged 32 to 46 Years with Incident Coronary Heart Disease and Death. JAMA Cardiol..

[26] Ceponiene I., Nakanishi R., Osawa K., Kanisawa M., Nezarat N., Rahmani S., Kissel K., Kim M., Jayawardena E., Broersen A. (2018). Coronary Artery Calcium Progression Is Associated with Coronary Plaque Volume Progression: Results From a Quantitative Semiautomated Coronary Artery Plaque Analysis. JACC Cardiovasc. Imaging.

[27] Tramontano L., Punzo B., Clemente A., Seitun S., Saba L., Bossone E., Maffei E., Cavaliere C., Cademartiri F. (2022). Prognostic Value of Coronary Calcium Score in Asymptomatic Individuals: A Systematic Review. J. Clin. Med..

[28] Peters S.A., Bakker M., den Ruijter H.M., Bots M.L. (2012). Added value of CAC in risk stratification for cardiovascular events: A systematic review. Eur. J. Clin. Investig..

[29] Greenland P., LaBree L., Azen S.P., Doherty T.M., Detrano R.C. (2004). Coronary artery calcium score combined with Framingham score for risk prediction in asymptomatic individuals. JAMA.

[30] Erbel R., Möhlenkamp S., Moebus S., Schmermund A., Lehmann N., Stang A., Dragano N., Grönemeyer D., Seibel R., Kälsch H. (2010). Heinz Nixdorf Recall Study Investigative Group. Coronary risk stratification, discrimination, and reclassification improvement based on quantification of subclinical coronary atherosclerosis: The Heinz Nixdorf Recall study. J. Am. Coll. Cardiol..

[31] McClelland R.L., Jorgensen N.W., Budoff M., Blaha M.J., Post W.S., Kronmal R.A., Bild D.E., Shea S., Liu K., Watson K.E. (2015). 10-Year Coronary Heart Disease Risk Prediction Using Coronary Artery Calcium and Traditional Risk Factors: Derivation in the MESA (Multi-Ethnic Study of Atherosclerosis) With Validation in the HNR (Heinz Nixdorf Recall) Study and the DHS (Dallas Heart Study). J. Am. Coll. Cardiol..

[32] Shaw L.J., Min J.K., Nasir K., Xie J.X., Berman D.S., Miedema M.D., Whelton S.P., Dardari Z.A., Rozanski A., Rumberger J. (2018). Sex differences in calcified plaque and long-term cardiovascular mortality: Observations from the CAC Consortium. Eur. Heart J..

[33] Wong N.D., Cordola Hsu A.R., Rozanski A., Shaw L.J., Whelton S.P., Budoff M.J., Nasir K., Miedema M.D., Rumberger J., Blaha M.J. (2020). Sex Differences in Coronary Artery Calcium and Mortality from Coronary Heart Disease, Cardiovascular Disease, and All Causes in Adults With Diabetes: The Coronary Calcium Consortium. Diabetes Care.

[34] Kavousi M., Desai C.S., Ayers C., Blumenthal R.S., Budoff M.J., Mahabadi A.A., Ikram M.A., van der Lugt A., Hofman A., Erbel R. (2016). Prevalence and Prognostic Implications of Coronary Artery Calcification in Low-Risk Women: A Meta-analysis. JAMA.

[35] Valenti V., Ó Hartaigh B., Heo R., Cho I., Schulman-Marcus J., Gransar H., Truong Q.A., Shaw L.J., Knapper J., Kelkar A.A. (2015). A 15-Year Warranty Period for Asymptomatic Individuals Without Coronary Artery Calcium: A Prospective Follow-Up of 9715 Individuals. JACC Cardiovasc. Imaging.

[36] Yeboah J., McClelland R.L., Polonsky T.S., Burke G.L., Sibley C.T., O’Leary D., Carr J.J., Goff D.C., Greenland P., Herrington D.M. (2012). Comparison of novel risk markers for improvement in cardiovascular risk assessment in intermediate-risk individuals. JAMA.

[37] Budoff M.J., Mayrhofer T., Ferencik M., Bittner D., Lee K.L., Lu M.T., Coles A., Jang J., Krishnam M., Douglas P.S. (2017). PROMISE Investigators. Prognostic Value of Coronary Artery Calcium in the PROMISE Study (Prospective Multicenter Imaging Study for Evaluation of Chest Pain). Circulation.

[38] Lo-Kioeng-Shioe M.S., Rijlaarsdam-Hermsen D., van Domburg R.T., Hadamitzky M., Lima J.A.C., Hoeks S.E., Deckers J.W. (2020). Prognostic value of coronary artery calcium score in symptomatic individuals: A meta-analysis of 34,000 subjects. Int. J. Cardiol..

[39] Mortensen M.B., Dzaye O., Steffensen F.H., Bøtker H.E., Jensen J.M., Rønnow Sand N.P., Kragholm K.H., Sørensen H.T., Leipsic J., Mæng M. (2020). Impact of Plaque Burden Versus Stenosis on Ischemic Events in Patients with Coronary Atherosclerosis. J. Am. Coll. Cardiol..

[40] Agha A.M., Pacor J., Grandhi G.R., Mszar R., Khan S.U., Parikh R., Agrawal T., Burt J., Blankstein R., Blaha M.J. (2022). The Prognostic Value of CAC Zero Among Individuals Presenting with Chest Pain: A Meta-Analysis. JACC Cardiovasc. Imaging.

[41] Villines T.C., Hulten E.A., Shaw L.J., Goyal M., Dunning A., Achenbach S., Al-Mallah M., Berman D.S., Budoff M.J., Cademartiri F. (2011). CONFIRM Registry Investigators. Prevalence and severity of coronary artery disease and adverse events among symptomatic patients with coronary artery calcification scores of zero undergoing coronary computed tomography angiography: Results from the CONFIRM (Coronary CT Angiography Evaluation for Clinical Outcomes: An International Multicenter) registry. J. Am. Coll. Cardiol..

[42] Mortensen M.B., Gaur S., Frimmer A., Bøtker H.E., Sørensen H.T., Kragholm K.H., Niels Peter S.R., Steffensen F.H., Jensen R.V., Mæng M. (2022). Association of Age with the Diagnostic Value of Coronary Artery Calcium Score for Ruling Out Coronary Stenosis in Symptomatic Patients. JAMA Cardiol..

[43] Leber A.W., Knez A., White C.W., Becker A., von Ziegler F., Muehling O., Becker C., Reiser M., Steinbeck G., Boekstegers P. (2003). Composition of coronary atherosclerotic plaques in patients with acute myocardial infarction and stable angina pectoris determined by contrast-enhanced multislice computed tomography. Am. J. Cardiol..

[44] Shemesh J., Apter S., Itzchak Y., Motro M. (2003). Coronary calcification compared in patients with acute versus in those with chronic coronary events by using dual-sector spiral CT. Radiology.

[45] Pugliese L., Spiritigliozzi L., Di Tosto F., Ricci F., Cavallo A.U., Di Donna C., De Stasio V., Presicce M., Benelli L., D’Errico F. (2020). Association of plaque calcification pattern and attenuation with instability features and coronary stenosis and calcification grade. Atherosclerosis.

[46] Houslay E.S., Cowell S.J., Prescott R.J., Reid J., Burton J., Northridge D.B., Boon N.A., Newby D.E. (2006). Scottish Aortic Stenosis and Lipid Lowering Therapy, Impact on Regression trial Investigators. Progressive coronary calcification despite intensive lipid-lowering treatment: A randomised controlled trial. Heart.

[47] Henein M., Granåsen G., Wiklund U., Schmermund A., Guerci A., Erbel R., Raggi P. (2015). High dose and long-term statin therapy accelerate coronary artery calcification. Int. J. Cardiol..

[48] Henein M.Y., Owen A. (2011). Statins moderate coronary stenoses but not coronary calcification: Results from meta-analyses. Int. J. Cardiol..

[49] Wang Y., Osborne M.T., Tung B., Li M., Li Y. (2018). Imaging Cardiovascular Calcification. J. Am. Heart Assoc..

[50] van Rosendael A.R., Narula J., Lin F.Y., van den Hoogen I.J., Gianni U., Al Hussein Alawamlh O., Dunham P.C., Peña J.M., Lee S.E., Andreini D. (2020). Association of High-Density Calcified 1K Plaque with Risk of Acute Coronary Syndrome. JAMA Cardiol..

[51] Criqui M.H., Denenberg J.O., Ix J.H., McClelland R.L., Wassel C.L., Rifkin D.E., Carr J.J., Budoff M.J., Allison M.A. (2014). Calcium density of coronary artery plaque and risk of incident cardiovascular events. JAMA.

[52] Iacobellis G. (2022). Epicardial adipose tissue in contemporary cardiology. Nat. Rev. Cardiol..

[53] Iacobellis G., Bianco A.C. (2011). Epicardial adipose tissue: Emerging physiological, pathophysiological and clinical features. Trends Endocrinol. Metab..

[54] Antonopoulos A.S., Antoniades C. (2017). The role of epicardial adipose tissue in cardiac biology: Classic concepts and emerging roles. J. Physiol..

[55] Packer M. (2018). Epicardial Adipose Tissue May Mediate Deleterious Effects of Obesity and Inflammation on the Myocardium. J. Am. Coll. Cardiol..

[56] Bos D., Leening M.J.G. (2018). Leveraging the coronary calcium scan beyond the coronary calcium score. Eur. Radiol..

[57] Goeller M., Achenbach S., Marwan M., Doris M.K., Cadet S., Commandeur F., Chen X., Slomka P.J., Gransar H., Cao J.J. (2018). Epicardial adipose tissue density and volume are related to subclinical atherosclerosis, inflammation and major adverse cardiac events in asymptomatic subjects. J. Cardiovasc. Comput. Tomogr..

[58] Bos D., Shahzad R., van Walsum T., van Vliet L.J., Franco O.H., Hofman A., Niessen W.J., Vernooij M.W., van der Lugt A. (2015). Epicardial fat volume is related to atherosclerotic calcification in multiple vessel beds. Eur. Heart J. Cardiovasc. Imaging.

[59] Mancio J., Pinheiro M., Ferreira W., Carvalho M., Barros A., Ferreira N., Vouga L., Ribeiro V.G., Leite-Moreira A., Falcao-Pires I. (2017). Gender differences in the association of epicardial adipose tissue and coronary artery calcification: EPICHEART study: EAT and coronary calcification by gender. Int. J. Cardiol..

[60] Yerramasu A., Dey D., Venuraju S., Anand D.V., Atwal S., Corder R., Berman D.S., Lahiri A. (2012). Increased volume of epicardial fat is an independent risk factor for accelerated progression of sub-clinical coronary atherosclerosis. Atherosclerosis.

[61] Nakanishi R., Rajani R., Cheng V.Y., Gransar H., Nakazato R., Shmilovich H., Otaki Y., Hayes S.W., Thomson L.E., Friedman J.D. (2011). Increase in epicardial fat volume is associated with greater coronary artery calcification progression in subjects at intermediate risk by coronary calcium score: A serial study using non-contrast cardiac CT. Atherosclerosis.

[62] Mahabadi A.A., Lehmann N., Kälsch H., Robens T., Bauer M., Dykun I., Budde T., Moebus S., Jöckel K.H., Erbel R. (2014). Association of epicardial adipose tissue with progression of coronary artery calcification is more pronounced in the early phase of atherosclerosis: Results from the Heinz Nixdorf recall study. JACC Cardiovasc. Imaging.

[63] Alexopoulos N., McLean D.S., Janik M., Arepalli C.D., Stillman A.E., Raggi P. (2010). Epicardial adipose tissue and coronary artery plaque characteristics. Atherosclerosis.

[64] Oka T., Yamamoto H., Ohashi N., Kitagawa T., Kunita E., Utsunomiya H., Yamazato R., Urabe Y., Horiguchi J., Awai K. (2012). Association between epicardial adipose tissue volume and characteristics of non-calcified plaques assessed by coronary computed tomographic angiography. Int. J. Cardiol..

[65] Bachar G.N., Dicker D., Kornowski R., Atar E. (2012). Epicardial adipose tissue as a predictor of coronary artery disease in asymptomatic subjects. Am. J. Cardiol..

[66] Jin X., Gao B., Zheng J., Wu X., Zhang N., Zhu L., Zhu X., Xie J., Wang Z., Tong G. (2023). Impact of epicardial adipose tissue volume on hemodynamically significant coronary artery disease in Chinese patients with known or suspected coronary artery disease. Front. Cardiovasc. Med..

[67] Tesche C., Bauer M.J., Straube F., Rogowski S., Baumann S., Renker M., Fink N., Schoepf U.J., Hoffmann E., Ebersberger U. (2022). Association of epicardial adipose tissue with coronary CT angiography plaque parameters on cardiovascular outcome in patients with and without diabetes mellitus. Atherosclerosis.

[68] Ito T., Suzuki Y., Ehara M., Matsuo H., Teramoto T., Terashima M., Nasu K., Kinoshita Y., Tsuchikane E., Suzuki T. (2013). Impact of epicardial fat volume on coronary artery disease in symptomatic patients with a zero calcium score. Int. J. Cardiol..

[69] Mancio J., Azevedo D., Saraiva F., Azevedo A.I., Pires-Morais G., Leite-Moreira A., Falcao-Pires I., Lunet N., Bettencourt N. (2018). Epicardial adipose tissue volume assessed by computed tomography and coronary artery disease: A systematic review and meta-analysis. Eur. Heart J. Cardiovasc. Imaging.

[70] Glaser R., Selzer F., Faxon D.P., Laskey W.K., Cohen H.A., Slater J., Detre K.M., Wilensky R.L. (2005). Clinical progression of incidental, asymptomatic lesions discovered during culprit vessel coronary intervention. Circulation.

[71] Stone G.W., Maehara A., Lansky A.J., de Bruyne B., Cristea E., Mintz G.S., Mehran R., McPherson J., Farhat N., Marso S.P. (2011). PROSPECT Investigators. A prospective natural-history study of coronary atherosclerosis. N. Engl. J. Med..

[72] Uren N.G., Melin J.A., De Bruyne B., Wijns W., Baudhuin T., Camici P.G. (1994). Relation between myocardial blood flow and the severity of coronary-artery stenosis. N. Engl. J. Med..

[73] Kochar M., Arsanjani R., Raman S.V., Shaw L.J., Berman D.S., Min J.K. (2012). Identifying and redefining stenosis by CT angiography. Cardiol. Clin..

[74] Cury R.C., Abbara S., Achenbach S., Agatston A., Berman D.S., Budoff M.J., Dill K.E., Jacobs J.E., Maroules C.D., Rubin G.D. (2016). CAD-RADS™: Coronary Artery Disease—Reporting and Data System: An Expert Consensus Document of the Society of Cardiovascular Computed Tomography (SCCT), the American College of Radiology (ACR) and the North American Society for Cardiovascular Imaging (NASCI). Endorsed by the American College of Cardiology. J. Am. Coll. Radiol..

[75] Hamon M., Biondi-Zoccai G.G., Malagutti P., Agostoni P., Morello R., Valgimigli M., Hamon M. (2006). Diagnostic performance of multislice spiral computed tomography of coronary arteries as compared with conventional invasive coronary angiography: A meta-analysis. J. Am. Coll. Cardiol..

[76] Miller J.M., Rochitte C.E., Dewey M., Arbab-Zadeh A., Niinuma H., Gottlieb I., Paul N., Clouse M.E., Shapiro E.P., Hoe J. (2008). Diagnostic performance of coronary angiography by 64-row CT. N. Engl. J. Med..

[77] Chao S.P., Law W.Y., Kuo C.J., Hung H.F., Cheng J.J., Lo H.M., Shyu K.G. (2010). The diagnostic accuracy of 256-row computed tomographic angiography compared with invasive coronary angiography in patients with suspected coronary artery disease. Eur. Heart J..

[78] Budoff M.J., Dowe D., Jollis J.G., Gitter M., Sutherland J., Halamert E., Scherer M., Bellinger R., Martin A., Benton R. (2008). Diagnostic performance of 64-multidetector row coronary computed tomographic angiography for evaluation of coronary artery stenosis in individuals without known coronary artery disease: Results from the prospective multicenter ACCURACY (Assessment by Coronary Computed Tomographic Angiography of Individuals Undergoing Invasive Coronary Angiography) trial. J. Am. Coll. Cardiol..

[79] Meijboom W.B., Meijs M.F., Schuijf J.D., Cramer M.J., Mollet N.R., van Mieghem C.A., Nieman K., van Werkhoven J.M., Pundziute G., Weustink A.C. (2008). Diagnostic accuracy of 64-slice computed tomography coronary angiography: A prospective, multicenter, multivendor study. J. Am. Coll. Cardiol..

[80] Dewey M., Rief M., Martus P., Kendziora B., Feger S., Dreger H., Priem S., Knebel F., Böhm M., Schlattmann P. (2016). Evaluation of computed tomography in patients with atypical angina or chest pain clinically referred for invasive coronary angiography: Randomised controlled trial. BMJ.

[81] Chang H.J., Lin F.Y., Gebow D., An H.Y., Andreini D., Bathina R., Baggiano A., Beltrama V., Cerci R., Choi E.Y. (2019). Selective Referral Using CCTA Versus Direct Referral for Individuals Referred to Invasive Coronary Angiography for Suspected CAD: A Randomized, Controlled, Open-Label Trial. JACC Cardiovasc. Imaging.

[82] Maurovich-Horvat P., Bosserdt M., Kofoed K.F., Rieckmann N., Benedek T., Donnelly P., Rodriguez-Palomares J., Erglis A., Štěchovský C., DISCHARGE Trial Group (2022). CT or Invasive Coronary Angiography in Stable Chest Pain. N. Engl. J. Med..

[83] Nørgaard B.L., Leipsic J., Achenbach S. (2018). Coronary CT Angiography to Guide Treatment Decision Making: Lessons From the SYNTAX II Trial. J. Am. Coll. Cardiol..

[84] Shalev A., Nakazato R., Arsanjani R., Nakanishi R., Park H.B., Otaki Y., Cheng V.Y., Gransar H., LaBounty T.M., Hayes S.W. (2016). SYNTAX Score Derived from Coronary CT Angiography for Prediction of Complex Percutaneous Coronary Interventions. Acad. Radiol..

[85] Suh Y.J., Han K., Chang S., Kim J.Y., Im D.J., Hong Y.J., Lee H.J., Hur J., Kim Y.J., Choi B.W. (2017). SYNTAX score based on coronary computed tomography angiography may have a prognostic value in patients with complex coronary artery disease: An observational study from a retrospective cohort. Medicine.

[86] Collet C., Onuma Y., Andreini D., Sonck J., Pompilio G., Mushtaq S., La Meir M., Miyazaki Y., de Mey J., Gaemperli O. (2018). Coronary computed tomography angiography for heart team decision-making in multivessel coronary artery disease. Eur. Heart J..

[87] Farooq V., van Klaveren D., Steyerberg E.W., Meliga E., Vergouwe Y., Chieffo A., Kappetein A.P., Colombo A., Holmes D.R., Mack M. (2013). Anatomical and clinical characteristics to guide decision making between coronary artery bypass surgery and percutaneous coronary intervention for individual patients: Development and validation of SYNTAX score II. Lancet.

[88] Nielsen L.H., Ortner N., Nørgaard B.L., Achenbach S., Leipsic J., Abdulla J. (2014). The diagnostic accuracy and outcomes after coronary computed tomography angiography vs. conventional functional testing in patients with stable angina pectoris: A systematic review and meta-analysis. Eur. Heart J. Cardiovasc. Imaging.

[89] Arbab-Zadeh A., Di Carli M.F., Cerci R., George R.T., Chen M.Y., Dewey M., Niinuma H., Vavere A.L., Betoko A., Plotkin M. (2015). Accuracy of Computed Tomographic Angiography and Single-Photon Emission Computed Tomography-Acquired Myocardial Perfusion Imaging for the Diagnosis of Coronary Artery Disease. Circ. Cardiovasc. Imaging.

[90] Danad I., Szymonifka J., Twisk J.W.R., Norgaard B.L., Zarins C.K., Knaapen P., Min J.K. (2017). Diagnostic performance of cardiac imaging methods to diagnose ischaemia-causing coronary artery disease when directly compared with fractional flow reserve as a reference standard: A meta-analysis. Eur. Heart J..

[91] Hollander J.E., Gatsonis C., Greco E.M., Snyder B.S., Chang A.M., Miller C.D., Singh H., Litt H.I. (2016). Coronary Computed Tomography Angiography Versus Traditional Care: Comparison of One-Year Outcomes and Resource Use. Ann. Emerg. Med..

[92] Levsky J.M., Spevack D.M., Travin M.I., Menegus M.A., Huang P.W., Clark E.T., Kim C.W., Hirschhorn E., Freeman K.D., Tobin J.N. (2015). Coronary Computed Tomography Angiography Versus Radionuclide Myocardial Perfusion Imaging in Patients with Chest Pain Admitted to Telemetry: A Randomized Trial. Ann. Intern. Med..

[93] Uretsky S., Argulian E., Supariwala A., Agarwal S.K., El-Hayek G., Chavez P., Awan H., Jagarlamudi A., Puppala S.P., Cohen R. (2017). Comparative effectiveness of coronary CT angiography vs. stress cardiac imaging in patients following hospital admission for chest pain work-up: The Prospective First Evaluation in Chest Pain (PERFECT) Trial. J. Nucl. Cardiol..

[94] Hoffmann U., Truong Q.A., Schoenfeld D.A., Chou E.T., Woodard P.K., Nagurney J.T., Pope J.H., Hauser T.H., White C.S., Weiner S.G. (2012). ROMICAT-II Investigators. Coronary CT angiography versus standard evaluation in acute chest pain. N. Engl. J. Med..

[95] Linde J.J., Hove J.D., Sørgaard M., Kelbæk H., Jensen G.B., Kühl J.T., Hindsø L., Køber L., Nielsen W.B., Kofoed K.F. (2015). Long-Term Clinical Impact of Coronary CT Angiography in Patients with Recent Acute-Onset Chest Pain: The Randomized Controlled CATCH Trial. JACC Cardiovasc. Imaging.

[96] Hamilton-Craig C., Fifoot A., Hansen M., Pincus M., Chan J., Walters D.L., Branch K.R. (2014). Diagnostic performance and cost of CT angiography versus stress ECG--a randomized prospective study of suspected acute coronary syndrome chest pain in the emergency department (CT-COMPARE). Int. J. Cardiol..

[97] Douglas P.S., Hoffmann U., Patel M.R., Mark D.B., Al-Khalidi H.R., Cavanaugh B., Cole J., Dolor R.J., Fordyce C.B., Huang M. (2015). PROMISE Investigators. Outcomes of anatomical versus functional testing for coronary artery disease. N. Engl. J. Med..

[98] Min J.K., Koduru S., Dunning A.M., Cole J.H., Hines J.L., Greenwell D., Biga C., Fanning G., LaBounty T.M., Gomez M. (2012). Coronary CT angiography versus myocardial perfusion imaging for near-term quality of life, cost and radiation exposure: A prospective multicenter randomized pilot trial. J. Cardiovasc. Comput. Tomogr..

[99] Newby D.E., Adamson P.D., Berry C., Boon N.A., Dweck M.R., Flather M., Forbes J., Hunter A., Lewis S., SCOT-HEART Investigators (2018). Coronary CT Angiography and 5-Year Risk of Myocardial Infarction. N. Engl. J. Med..

[100] Lubbers M., Dedic A., Coenen A., Galema T., Akkerhuis J., Bruning T., Krenning B., Musters P., Ouhlous M., Liem A. (2016). Calcium imaging and selective computed tomography angiography in comparison to functional testing for suspected coronary artery disease: The multicentre, randomized CRESCENT trial. Eur. Heart J..

[101] McKavanagh P., Lusk L., Ball P.A., Verghis R.M., Agus A.M., Trinick T.R., Duly E., Walls G.M., Stevenson M., James B. (2015). A comparison of cardiac computerized tomography and exercise stress electrocardiogram test for the investigation of stable chest pain: The clinical results of the CAPP randomized prospective trial. Eur. Heart J. Cardiovasc. Imaging.

[102] Hwang I.C., Choi S.J., Choi J.E., Ko E.B., Suh J.K., Choi I., Kang H.J., Kim Y.J., Kim J.Y. (2017). Comparison of mid- to long-term clinical outcomes between anatomical testing and usual care in patients with suspected coronary artery disease: A meta-analysis of randomized trials. Clin. Cardiol..

[103] Burch R.A., Siddiqui T.A., Tou L.C., Turner K.B., Umair M. (2023). The Cost Effectiveness of Coronary CT Angiography and the Effective Utilization of CT-Fractional Flow Reserve in the Diagnosis of Coronary Artery Disease. J. Cardiovasc. Dev. Dis..

[104] Andreini D., Pontone G., Mushtaq S., Bartorelli A.L., Bertella E., Antonioli L., Formenti A., Cortinovis S., Veglia F., Annoni A. (2012). A long-term prognostic value of coronary CT angiography in suspected coronary artery disease. JACC Cardiovasc. Imaging.

[105] Min J.K., Shaw L.J., Devereux R.B., Okin P.M., Weinsaft J.W., Russo D.J., Lippolis N.J., Berman D.S., Callister T.Q. (2007). Prognostic value of multidetector coronary computed tomographic angiography for prediction of all-cause mortality. J. Am. Coll. Cardiol..

[106] Hadamitzky M., Achenbach S., Al-Mallah M., Berman D., Budoff M., Cademartiri F., Callister T., Chang H.J., Cheng V., Chinnaiyan K. (2013). CONFIRM Investigators. Optimized prognostic score for coronary computed tomographic angiography: Results from the CONFIRM registry (COronary CT Angiography EvaluatioN for Clinical Outcomes: An InteRnational Multicenter Registry). J. Am. Coll. Cardiol..

[107] Clerc O.F., Kaufmann B.P., Possner M., Liga R., Vontobel J., Mikulicic F., Gräni C., Benz D.C., Fuchs T.A., Stehli J. (2017). Long-term prognostic performance of low-dose coronary computed tomography angiography with prospective electrocardiogram triggering. Eur. Radiol..

[108] Nielsen L.H., Bøtker H.E., Sørensen H.T., Schmidt M., Pedersen L., Sand N.P., Jensen J.M., Steffensen F.H., Tilsted H.H., Bøttcher M. (2017). Prognostic assessment of stable coronary artery disease as determined by coronary computed tomography angiography: A Danish multicentre cohort study. Eur. Heart J..

[109] Ostrom M.P., Gopal A., Ahmadi N., Nasir K., Yang E., Kakadiaris I., Flores F., Mao S.S., Budoff M.J. (2008). Mortality incidence and the severity of coronary atherosclerosis assessed by computed tomography angiography. J. Am. Coll. Cardiol..

[110] Bittner D.O., Mayrhofer T., Budoff M., Szilveszter B., Foldyna B., Hallett T.R., Ivanov A., Janjua S., Meyersohn N.M., Staziaki P.V. (2020). PROMISE Investigators. Prognostic Value of Coronary CTA in Stable Chest Pain: CAD-RADS, CAC, and Cardiovascular Events in PROMISE. JACC Cardiovasc. Imaging.

[111] Hoffmann U., Ferencik M., Udelson J.E., Picard M.H., Truong Q.A., Patel M.R., Huang M., Pencina M., Mark D.B., Heitner J.F. (2017). PROMISE Investigators. Prognostic Value of Noninvasive Cardiovascular Testing in Patients with Stable Chest Pain: Insights From the PROMISE Trial (Prospective Multicenter Imaging Study for Evaluation of Chest Pain). Circulation.

[112] Cantoni V., Green R., Acampa W., Petretta M., Bonaduce D., Salvatore M., Cuocolo A. (2016). Long-term prognostic value of stress myocardial perfusion imaging and coronary computed tomography angiography: A meta-analysis. J. Nucl. Cardiol..

[113] Hoffmann U., Bamberg F., Chae C.U., Nichols J.H., Rogers I.S., Seneviratne S.K., Truong Q.A., Cury R.C., Abbara S., Shapiro M.D. (2009). Coronary computed tomography angiography for early triage of patients with acute chest pain: The ROMICAT (Rule Out Myocardial Infarction using Computer Assisted Tomography) trial. J. Am. Coll. Cardiol..

[114] Smulders M.W., Jaarsma C., Nelemans P.J., Bekkers S.C.A.M., Bucerius J., Leiner T., Crijns H.J.G.M., Wildberger J.E., Schalla S. (2017). Comparison of the prognostic value of negative non-invasive cardiac investigations in patients with suspected or known coronary artery disease-a meta-analysis. Eur. Heart J. Cardiovasc. Imaging.

[115] Hadamitzky M., Täubert S., Deseive S., Byrne R.A., Martinoff S., Schömig A., Hausleiter J. (2013). Prognostic value of coronary computed tomography angiography during 5 years of follow-up in patients with suspected coronary artery disease. Eur. Heart J..

[116] Finck T., Hardenberg J., Will A., Hendrich E., Haller B., Martinoff S., Hausleiter J., Hadamitzky M. (2019). 10-Year Follow-Up After Coronary Computed Tomography Angiography in Patients with Suspected Coronary Artery Disease. JACC Cardiovasc. Imaging.

[117] Jeremias A., Kirtane A.J., Stone G.W. (2017). A Test in Context: Fractional Flow Reserve: Accuracy, Prognostic Implications, and Limitations. J. Am. Coll. Cardiol..

[118] Reynolds H.R., Shaw L.J., Min J.K., Page C.B., Berman D.S., Chaitman B.R., Picard M.H., Kwong R.Y., O’Brien S.M., Huang Z. (2021). Outcomes in the ISCHEMIA Trial Based on Coronary Artery Disease and Ischemia Severity. Circulation.

[119] Bech G.J., De Bruyne B., Pijls N.H., de Muinck E.D., Hoorntje J.C., Escaned J., Stella P.R., Boersma E., Bartunek J., Koolen J.J. (2001). Fractional flow reserve to determine the appropriateness of angioplasty in moderate coronary stenosis: A randomized trial. Circulation.

[120] Tonino P.A., De Bruyne B., Pijls N.H., Siebert U., Ikeno F., van’t Veer M., Klauss V., Manoharan G., Engstrøm T., Oldroyd K.G. (2009). FAME Study Investigators. Fractional flow reserve versus angiography for guiding percutaneous coronary intervention. N. Engl. J. Med..

[121] Seitun S., Clemente A., De Lorenzi C., Benenati S., Chiappino D., Mantini C., Sakellarios A.I., Cademartiri F., Bezante G.P., Porto I. (2020). Cardiac CT perfusion and FFR_CTA_: Pathophysiological features in ischemic heart disease. Cardiovasc. Diagn. Ther..

[122] Dai N., Zhang X., Zhang Y., Hou L., Li W., Fan B., Zhang T., Xu Y. (2016). Enhanced diagnostic utility achieved by myocardial blood analysis: A meta-analysis of noninvasive cardiac imaging in the detection of functional coronary artery disease. Int. J. Cardiol..

[123] Gonzalez J.A., Lipinski M.J., Flors L., Shaw P.W., Kramer C.M., Salerno M. (2015). Meta-Analysis of Diagnostic Performance of Coronary Computed Tomography Angiography, Computed Tomography Perfusion, and Computed Tomography-Fractional Flow Reserve in Functional Myocardial Ischemia Assessment Versus Invasive Fractional Flow Reserve. Am. J. Cardiol..

[124] Zhuang B., Wang S., Zhao S., Lu M. (2020). Computed tomography angiography-derived fractional flow reserve (CT-FFR) for the detection of myocardial ischemia with invasive fractional flow reserve as reference: Systematic review and meta-analysis. Eur. Radiol..

[125] Takx R.A., Blomberg B.A., El Aidi H., Habets J., de Jong P.A., Nagel E., Hoffmann U., Leiner T. (2015). Diagnostic accuracy of stress myocardial perfusion imaging compared to invasive coronary angiography with fractional flow reserve meta-analysis. Circ. Cardiovasc. Imaging.

[126] Fairbairn T.A., Nieman K., Akasaka T., Nørgaard B.L., Berman D.S., Raff G., Hurwitz-Koweek L.M., Pontone G., Kawasaki T., Sand N.P. (2018). Real-world clinical utility and impact on clinical decision-making of coronary computed tomography angiography-derived fractional flow reserve: Lessons from the ADVANCE Registry. Eur. Heart J..

[127] Nørgaard B.L., Leipsic J., Gaur S., Seneviratne S., Ko B.S., Ito H., Jensen J.M., Mauri L., De Bruyne B., Bezerra H. (2014). NXT Trial Study Group. Diagnostic performance of noninvasive fractional flow reserve derived from coronary computed tomography angiography in suspected coronary artery disease: The NXT trial (Analysis of Coronary Blood Flow Using CT Angiography: Next Steps). J. Am. Coll. Cardiol..

[128] Driessen R.S., Danad I., Stuijfzand W.J., Raijmakers P.G., Schumacher S.P., van Diemen P.A., Leipsic J.A., Knuuti J., Underwood S.R., van de Ven P.M. (2019). Comparison of Coronary Computed Tomography Angiography, Fractional Flow Reserve, and Perfusion Imaging for Ischemia Diagnosis. J. Am. Coll. Cardiol..

[129] Patel M.R., Nørgaard B.L., Fairbairn T.A., Nieman K., Akasaka T., Berman D.S., Raff G.L., Hurwitz Koweek L.M., Pontone G., Kawasaki T. (2020). 1-Year Impact on Medical Practice and Clinical Outcomes of FFR_CT_: The ADVANCE Registry. JACC Cardiovasc. Imaging.

[130] Ihdayhid A.R., Norgaard B.L., Gaur S., Leipsic J., Nerlekar N., Osawa K., Miyoshi T., Jensen J.M., Kimura T., Shiomi H. (2019). Prognostic Value and Risk Continuum of Noninvasive Fractional Flow Reserve Derived from Coronary CT Angiography. Radiology.

[131] Sonck J., Nagumo S., Norgaard B.L., Otake H., Ko B., Zhang J., Mizukami T., Maeng M., Andreini D., Takahashi Y. (2022). Clinical Validation of a Virtual Planner for Coronary Interventions Based on Coronary CT Angiography. JACC Cardiovasc. Imaging.

[132] Li S.J., Ge Z., Kan J., Zhang J.J., Ye F., Kwan T.W., Santoso T., Yang S., Sheiban I., Qian X.S. (2017). Cutoff Value and Long-Term Prediction of Clinical Events by FFR Measured Immediately After Implantation of a Drug-Eluting Stent in Patients with Coronary Artery Disease: 1- to 3-Year Results From the DKCRUSH VII Registry Study. JACC Cardiovasc. Interv..

[133] Piroth Z., Toth G.G., Tonino P.A.L., Barbato E., Aghlmandi S., Curzen N., Rioufol G., Pijls N.H.J., Fearon W.F., Jüni P. (2017). Prognostic Value of Fractional Flow Reserve Measured Immediately After Drug-Eluting Stent Implantation. Circ. Cardiovasc. Interv..

[134] Mileva N., Ohashi H., Paolisso P., Leipsic J., Mizukami T., Sonck J., Norgaard B.L., Otake H., Ko B., Maeng M. (2023). Relationship between coronary volume, myocardial mass, and post-PCI fractional flow reserve. Catheter. Cardiovasc. Interv..

[135] Ide S., Sumitsuji S., Yamaguchi O., Sakata Y. (2017). Cardiac computed tomography-derived myocardial mass at risk using the Voronoi-based segmentation algorithm: A histological validation study. J. Cardiovasc. Comput. Tomogr..

[136] Zhou T., Wang X., Wu T., Yang Z., Li S., Li Y., He F., Zhang M., Yang C., Jia S. (2021). Clinical application of computed tomography angiography and fractional flow reserve computed tomography in patients with coronary artery disease: A meta-analysis based on pre- and post-test probability. Eur. J. Radiol..

[137] Ko B.S., Cameron J.D., Meredith I.T., Leung M., Antonis P.R., Nasis A., Crossett M., Hope S.A., Lehman S.J., Troupis J. (2012). Computed tomography stress myocardial perfusion imaging in patients considered for revascularization: A comparison with fractional flow reserve. Eur. Heart J..

[138] Bettencourt N., Chiribiri A., Schuster A., Ferreira N., Sampaio F., Pires-Morais G., Santos L., Melica B., Rodrigues A., Braga P. (2013). Direct comparison of cardiac magnetic resonance and multidetector computed tomography stress-rest perfusion imaging for detection of coronary artery disease. J. Am. Coll. Cardiol..

[139] Rief M., Zimmermann E., Stenzel F., Martus P., Stangl K., Greupner J., Knebel F., Kranz A., Schlattmann P., Laule M. (2013). Computed tomography angiography and myocardial computed tomography perfusion in patients with coronary stents: Prospective intraindividual comparison with conventional coronary angiography. J. Am. Coll. Cardiol..

[140] Rochitte C.E., George R.T., Chen M.Y., Arbab-Zadeh A., Dewey M., Miller J.M., Niinuma H., Yoshioka K., Kitagawa K., Nakamori S. (2014). Computed tomography angiography and perfusion to assess coronary artery stenosis causing perfusion defects by single photon emission computed tomography: The CORE320 study. Eur. Heart J..

[141] De Cecco C.N., Harris B.S., Schoepf U.J., Silverman J.R., McWhite C.B., Krazinski A.W., Bayer R.R., Meinel F.G. (2014). Incremental value of pharmacological stress cardiac dual-energy CT over coronary CT angiography alone for the assessment of coronary artery disease in a high-risk population. AJR Am. J. Roentgenol..

[142] Wichmann J.L., Meinel F.G., Schoepf U.J., Lo G.G., Choe Y.H., Wang Y., Vliegenthart R., Varga-Szemes A., Muscogiuri G., Cannaò P.M. (2015). Absolute Versus Relative Myocardial Blood Flow by Dynamic CT Myocardial Perfusion Imaging in Patients with Anatomic Coronary Artery Disease. AJR Am. J. Roentgenol..

[143] Pontone G., Andreini D., Guaricci A.I., Baggiano A., Fazzari F., Guglielmo M., Muscogiuri G., Berzovini C.M., Pasquini A., Mushtaq S. (2019). Incremental Diagnostic Value of Stress Computed Tomography Myocardial Perfusion with Whole-Heart Coverage CT Scanner in Intermediate- to High-Risk Symptomatic Patients Suspected of Coronary Artery Disease. JACC Cardiovasc. Imaging.

[144] Danad I., Szymonifka J., Schulman-Marcus J., Min J.K. (2016). Static and dynamic assessment of myocardial perfusion by computed tomography. Eur. Heart J. Cardiovasc. Imaging.

[145] Lu M., Wang S., Sirajuddin A., Arai A.E., Zhao S. (2018). Dynamic stress computed tomography myocardial perfusion for detecting myocardial ischemia: A systematic review and meta-analysis. Int. J. Cardiol..

[146] Lubbers M., Coenen A., Kofflard M., Bruning T., Kietselaer B., Galema T., Kock M., Niezen A., Das M., van Gent M. (2018). Comprehensive Cardiac CT With Myocardial Perfusion Imaging Versus Functional Testing in Suspected Coronary Artery Disease: The Multicenter, Randomized CRESCENT-II Trial. JACC Cardiovasc. Imaging.

[147] Nakamura S., Kitagawa K., Goto Y., Omori T., Kurita T., Yamada A., Takafuji M., Uno M., Dohi K., Sakuma H. (2019). Incremental Prognostic Value of Myocardial Blood Flow Quantified with Stress Dynamic Computed Tomography Perfusion Imaging. JACC Cardiovasc. Imaging.

[148] Yang J., Dou G., He B., Jin Q., Chen Z., Jing J., Di Carli M.F., Chen Y., Blankstein R. (2020). Stress Myocardial Blood Flow Ratio by Dynamic CT Perfusion Identifies Hemodynamically Significant CAD. JACC Cardiovasc. Imaging.

[149] Yang D.H., Kim Y.H., Roh J.H., Kang J.W., Ahn J.M., Kweon J., Lee J.B., Choi S.H., Shin E.S., Park D.W. (2017). Diagnostic performance of on-site CT-derived fractional flow reserve versus CT perfusion. Eur. Heart J. Cardiovasc. Imaging.

[150] Coenen A., Rossi A., Lubbers M.M., Kurata A., Kono A.K., Chelu R.G., Segreto S., Dijkshoorn M.L., Wragg A., van Geuns R.M. (2017). Integrating CT Myocardial Perfusion and CT-FFR in the Work-Up of Coronary Artery Disease. JACC Cardiovasc. Imaging.

[151] Pontone G., Baggiano A., Andreini D., Guaricci A.I., Guglielmo M., Muscogiuri G., Fusini L., Fazzari F., Mushtaq S., Conte E. (2019). Stress Computed Tomography Perfusion Versus Fractional Flow Reserve CT Derived in Suspected Coronary Artery Disease: The PERFECTION Study. JACC Cardiovasc. Imaging.

[152] Glagov S., Bassiouny H.S., Sakaguchi Y., Goudet C.A., Vito R.P. (1997). Mechanical determinants of plaque modeling, remodeling and disruption. Atherosclerosis.

[153] Ahmadi A., Argulian E., Leipsic J., Newby D.E., Narula J. (2019). From Subclinical Atherosclerosis to Plaque Progression and Acute Coronary Events: JACC State-of-the-Art Review. J. Am. Coll. Cardiol..

[154] Bentzon J.F., Otsuka F., Virmani R., Falk E. (2014). Mechanisms of plaque formation and rupture. Circ. Res..

[155] Shah P.K. (2002). Pathophysiology of coronary thrombosis: Role of plaque rupture and plaque erosion. Prog. Cardiovasc. Dis..

[156] Burke A.P., Kolodgie F.D., Farb A., Weber D., Virmani R. (2002). Morphological predictors of arterial remodeling in coronary atherosclerosis. Circulation.

[157] Virmani R., Burke A.P., Farb A., Kolodgie F.D. (2006). Pathology of the vulnerable plaque. J. Am. Coll. Cardiol..

[158] Ehara S., Kobayashi Y., Yoshiyama M., Shimada K., Shimada Y., Fukuda D., Nakamura Y., Yamashita H., Yamagishi H., Takeuchi K. (2004). Spotty calcification typifies the culprit plaque in patients with acute myocardial infarction: An intravascular ultrasound study. Circulation.

[159] Baumann S., Renker M., Meinel F.G., Wichmann J.L., Fuller S.R., Bayer R.R., Schoepf U.J., Steinberg D.H. (2015). Computed tomography imaging of coronary artery plaque: Characterization and prognosis. Radiol. Clin. North Am..

[160] Schuhbaeck A., Dey D., Otaki Y., Slomka P., Kral B.G., Achenbach S., Berman D.S., Fishman E.K., Lai S., Lai H. (2014). Interscan reproducibility of quantitative coronary plaque volume and composition from CT coronary angiography using an automated method. Eur. Radiol..

[161] Nakanishi R., Ceponiene I., Osawa K., Luo Y., Kanisawa M., Megowan N., Nezarat N., Rahmani S., Broersen A., Kitslaar P.H. (2016). Plaque progression assessed by a novel semi-automated quantitative plaque software on coronary computed tomography angiography between diabetes and non-diabetes patients: A propensity-score matching study. Atherosclerosis.

[162] van Rosendael A.R., Lin F.Y., Ma X., van den Hoogen I.J., Gianni U., Al Hussein O., Al’Aref S.J., Peña J.M., Andreini D., Al-Mallah M.H. (2020). Percent atheroma volume: Optimal variable to report whole-heart atherosclerotic plaque burden with coronary CTA, the PARADIGM study. J. Cardiovasc. Comput. Tomogr..

[163] Saremi F., Achenbach S. (2015). Coronary plaque characterization using CT. AJR Am. J. Roentgenol..

[164] Schlett C.L., Maurovich-Horvat P., Ferencik M., Alkadhi H., Stolzmann P., Scheffel H., Seifarth H., Nakano M., Do S., Vorpahl M. (2013). Histogram analysis of lipid-core plaques in coronary computed tomographic angiography: Ex vivo validation against histology. Investig. Radiol..

[165] Motoyama S., Kondo T., Sarai M., Sugiura A., Harigaya H., Sato T., Inoue K., Okumura M., Ishii J., Anno H. (2007). Multislice computed tomographic characteristics of coronary lesions in acute coronary syndromes. J. Am. Coll. Cardiol..

[166] Maurovich-Horvat P., Hoffmann U., Vorpahl M., Nakano M., Virmani R., Alkadhi H. (2010). The napkin-ring sign: CT signature of high-risk coronary plaques?. JACC Cardiovasc. Imaging.

[167] Narula J., Shaw L.J., Bax J.J., Min J.K., Chang H.J. (2020). Quantitative assessment of coronary plaque volume change related to triglyceride glucose index: The Progression of AtheRosclerotic PlAque DetermIned by Computed TomoGraphic Angiography IMaging (PARADIGM) registry. Cardiovasc. Diabetol..

[168] Petranovic M., Soni A., Bezzera H., Loureiro R., Sarwar A., Raffel C., Pomerantsev E., Jang I.K., Brady T.J., Achenbach S. (2009). Assessment of nonstenotic coronary lesions by 64-slice multidetector computed tomography in comparison to intravascular ultrasound: Evaluation of nonculprit coronary lesions. J. Cardiovasc. Comput. Tomogr..

[169] Enrico B., Suranyi P., Thilo C., Bonomo L., Costello P., Schoepf U.J. (2009). Coronary artery plaque formation at coronary CT angiography: Morphological analysis and relationship to hemodynamics. Eur. Radiol..

[170] Henzler T., Porubsky S., Kayed H., Harder N., Krissak U.R., Meyer M., Sueselbeck T., Marx A., Michaely H., Schoepf U.J. (2011). Attenuation-based characterization of coronary atherosclerotic plaque: Comparison of dual source and dual energy CT with single-source CT and histopathology. Eur. J. Radiol..

[171] Nakazato R., Shalev A., Doh J.H., Koo B.K., Dey D., Berman D.S., Min J.K. (2013). Quantification and characterisation of coronary artery plaque volume and adverse plaque features by coronary computed tomographic angiography: A direct comparison to intravascular ultrasound. Eur. Radiol..

[172] Doh J.H., Koo B.K., Nam C.W., Kim J.H., Min J.K., Nakazato R., Silalahi T., Prawira H., Choi H., Lee S.Y. (2014). Diagnostic value of coronary CT angiography in comparison with invasive coronary angiography and intravascular ultrasound in patients with intermediate coronary artery stenosis: Results from the prospective multicentre FIGURE-OUT (Functional Imaging criteria for GUiding REview of invasive coronary angiOgraphy, intravascular Ultrasound, and coronary computed Tomographic angiography) study. Eur. Heart J. Cardiovasc. Imaging.

[173] Conte E., Mushtaq S., Pontone G., Li Piani L., Ravagnani P., Galli S., Collet C., Sonck J., Di Odoardo L., Guglielmo M. (2020). Plaque quantification by coronary computed tomography angiography using intravascular ultrasound as a reference standard: A comparison between standard and last generation computed tomography scanners. Eur. Heart J. Cardiovasc. Imaging.

[174] Gao D., Ning N., Guo Y., Ning W., Niu X., Yang J. (2011). Computed tomography for detecting coronary artery plaques: A meta-analysis. Atherosclerosis.

[175] Fischer C., Hulten E., Belur P., Smith R., Voros S., Villines T.C. (2013). Coronary CT angiography versus intravascular ultrasound for estimation of coronary stenosis and atherosclerotic plaque burden: A meta-analysis. J. Cardiovasc. Comput. Tomogr..

[176] Voros S., Rinehart S., Qian Z., Vazquez G., Anderson H., Murrieta L., Wilmer C., Carlson H., Taylor K., Ballard W. (2011). Prospective validation of standardized, 3-dimensional, quantitative coronary computed tomographic plaque measurements using radiofrequency backscatter intravascular ultrasound as reference standard in intermediate coronary arterial lesions: Results from the ATLANTA (assessment of tissue characteristics, lesion morphology, and hemodynamics by angiography with fractional flow reserve, intravascular ultrasound and virtual histology, and noninvasive computed tomography in atherosclerotic plaques) I study. JACC Cardiovasc. Interv..

[177] Obaid D.R., Calvert P.A., Gopalan D., Parker R.A., Hoole S.P., West N.E., Goddard M., Rudd J.H., Bennett M.R. (2013). Atherosclerotic plaque composition and classification identified by coronary computed tomography: Assessment of computed tomography-generated plaque maps compared with virtual histology intravascular ultrasound and histology. Circ. Cardiovasc. Imaging.

[178] de Graaf M.A., Broersen A., Kitslaar P.H., Roos C.J., Dijkstra J., Lelieveldt B.P., Jukema J.W., Schalij M.J., Delgado V., Bax J.J. (2013). Automatic quantification and characterization of coronary atherosclerosis with computed tomography coronary angiography: Cross-correlation with intravascular ultrasound virtual histology. Int. J. Cardiovasc. Imaging.

[179] Bamberg F., Sommer W.H., Hoffmann V., Achenbach S., Nikolaou K., Conen D., Reiser M.F., Hoffmann U., Becker C.R. (2011). Meta-analysis and systematic review of the long-term predictive value of assessment of coronary atherosclerosis by contrast-enhanced coronary computed tomography angiography. J. Am. Coll. Cardiol..

[180] Min J.K., Chang H.J., Andreini D., Pontone G., Guglielmo M., Bax J.J., Knaapen P., Raman S.V., Chazal R.A., Freeman A.M. (2022). Coronary CTA plaque volume severity stages according to invasive coronary angiography and FFR. J. Cardiovasc. Comput. Tomogr..

[181] Vattay B., Borzsák S., Boussoussou M., Vecsey-Nagy M., Jermendy Á.L., Suhai F.I., Maurovich-Horvat P., Merkely B., Kolossvary M., Szilveszter B. (2022). Association between coronary plaque volume and myocardial ischemia detected by dynamic perfusion CT imaging. Front. Cardiovasc. Med..

[182] Deseive S., Kupke M., Straub R., Stocker T.J., Broersen A., Kitslaar P., Martinoff S., Massberg S., Hadamitzky M., Hausleiter J. (2021). Quantified coronary total plaque volume from computed tomography angiography provides superior 10-year risk stratification. Eur. Heart J. Cardiovasc. Imaging.

[183] Halon D.A., Lavi I., Barnett-Griness O., Rubinshtein R., Zafrir B., Azencot M., Lewis B.S. (2019). Plaque Morphology as Predictor of Late Plaque Events in Patients with Asymptomatic Type 2 Diabetes: A Long-Term Observational Study. JACC Cardiovasc. Imaging.

[184] Puri R., Nissen S.E., Shao M., Ballantyne C.M., Barter P.J., Chapman M.J., Erbel R., Libby P., Raichlen J.S., Uno K. (2013). Coronary atheroma volume and cardiovascular events during maximally intensive statin therapy. Eur. Heart J..

[185] Chow B.J., Wells G.A., Chen L., Yam Y., Galiwango P., Abraham A., Sheth T., Dennie C., Beanlands R.S., Ruddy T.D. (2010). Prognostic value of 64-slice cardiac computed tomography severity of coronary artery disease, coronary atherosclerosis, and left ventricular ejection fraction. J. Am. Coll. Cardiol..

[186] Hou Z.H., Lu B., Gao Y., Jiang S.L., Wang Y., Li W., Budoff M.J. (2012). Prognostic value of coronary CT angiography and calcium score for major adverse cardiac events in outpatients. JACC Cardiovasc. Imaging.

[187] Chang H.J., Lin F.Y., Lee S.E., Andreini D., Bax J., Cademartiri F., Chinnaiyan K., Chow B.J.W., Conte E., Cury R.C. (2018). Coronary Atherosclerotic Precursors of Acute Coronary Syndromes. J. Am. Coll. Cardiol..

[188] Hell M.M., Motwani M., Otaki Y., Cadet S., Gransar H., Miranda-Peats R., Valk J., Slomka P.J., Cheng V.Y., Rozanski A. (2017). Quantitative global plaque characteristics from coronary computed tomography angiography for the prediction of future cardiac mortality during long-term follow-up. Eur. Heart J. Cardiovasc. Imaging.

[189] Ahmadi A., Leipsic J., Øvrehus K.A., Gaur S., Bagiella E., Ko B., Dey D., LaRocca G., Jensen J.M., Bøtker H.E. (2018). Lesion-Specific and Vessel-Related Determinants of Fractional Flow Reserve Beyond Coronary Artery Stenosis. JACC Cardiovasc. Imaging.

[190] Bakhshi H., Meyghani Z., Kishi S., Magalhães T.A., Vavere A., Kitslaar P.H., George R.T., Niinuma H., Reiber J.H.C., Betoko A. (2019). Comparative Effectiveness of CT-Derived Atherosclerotic Plaque Metrics for Predicting Myocardial Ischemia. JACC Cardiovasc. Imaging.

[191] Benedek T., Gyöngyösi M., Benedek I. (2013). Multislice computed tomographic coronary angiography for quantitative assessment of culprit lesions in acute coronary syndromes. Can. J. Cardiol..

[192] Motoyama S., Ito H., Sarai M., Kondo T., Kawai H., Nagahara Y., Harigaya H., Kan S., Anno H., Takahashi H. (2015). Plaque Characterization by Coronary Computed Tomography Angiography and the Likelihood of Acute Coronary Events in Mid-Term Follow-Up. J. Am. Coll. Cardiol..

[193] Ferencik M., Mayrhofer T., Bittner D.O., Emami H., Puchner S.B., Lu M.T., Meyersohn N.M., Ivanov A.V., Adami E.C., Patel M.R. (2018). Use of High-Risk Coronary Atherosclerotic Plaque Detection for Risk Stratification of Patients with Stable Chest Pain: A Secondary Analysis of the PROMISE Randomized Clinical Trial. JAMA Cardiol..

[194] Williams M.C., Moss A.J., Dweck M., Adamson P.D., Alam S., Hunter A., Shah A.S.V., Pawade T., Weir-McCall J.R., Roditi G. (2019). Coronary Artery Plaque Characteristics Associated with Adverse Outcomes in the SCOT-HEART Study. J. Am. Coll. Cardiol..

[195] Otsuka K., Fukuda S., Tanaka A., Nakanishi K., Taguchi H., Yoshikawa J., Shimada K., Yoshiyama M. (2013). Napkin-ring sign on coronary CT angiography for the prediction of acute coronary syndrome. JACC Cardiovasc. Imaging.

[196] Puchner S.B., Liu T., Mayrhofer T., Truong Q.A., Lee H., Fleg J.L., Nagurney J.T., Udelson J.E., Hoffmann U., Ferencik M. (2014). High-risk plaque detected on coronary CT angiography predicts acute coronary syndromes independent of significant stenosis in acute chest pain: Results from the ROMICAT-II trial. J. Am. Coll. Cardiol..

[197] Erlinge D., Maehara A., Ben-Yehuda O., Bøtker H.E., Maeng M., Kjøller-Hansen L., Engstrøm T., Matsumura M., Crowley A., Dressler O. (2021). PROSPECT II Investigators. Identification of vulnerable plaques and patients by intracoronary near-infrared spectroscopy and ultrasound (PROSPECT II): A prospective natural history study. Lancet.

[198] Antonopoulos A.S., Sanna F., Sabharwal N., Thomas S., Oikonomou E.K., Herdman L., Margaritis M., Shirodaria C., Kampoli A.M., Akoumianakis I. (2017). Detecting human coronary inflammation by imaging perivascular fat. Sci. Transl. Med..

[199] Akoumianakis I., Antoniades C. (2017). The interplay between adipose tissue and the cardiovascular system: Is fat always bad?. Cardiovasc. Res..

[200] Antoniades C., Kotanidis C.P., Berman D.S. (2019). State-of-the-art review article. Atherosclerosis affecting fat: What can we learn by imaging perivascular adipose tissue?. J. Cardiovasc. Comput. Tomogr..

[201] Libby P. (2021). The changing landscape of atherosclerosis. Nature.

[202] Mazurek T., Kobylecka M., Zielenkiewicz M., Kurek A., Kochman J., Filipiak K.J., Mazurek K., Huczek Z., Królicki L., Opolski G. (2017). PET/CT evaluation of 18F-FDG uptake in pericoronary adipose tissue in patients with stable coronary artery disease: Independent predictor of atherosclerotic lesions’ formation?. J. Nucl. Cardiol..

[203] Kwiecinski J., Dey D., Cadet S., Lee S.E., Otaki Y., Huynh P.T., Doris M.K., Eisenberg E., Yun M., Jansen M.A. (2019). Peri-Coronary Adipose Tissue Density Is Associated With ^18^F-Sodium Fluoride Coronary Uptake in Stable Patients with High-Risk Plaques. JACC Cardiovasc. Imaging.

[204] Goeller M., Rahman Ihdayhid A., Cadet S., Lin A., Adams D., Thakur U., Yap G., Marwan M., Achenbach S., Damini D. (2020). Pericoronary adipose tissue and quantitative global non-calcified plaque characteristics from CT angiography do not differ in matched South Asian, East Asian and European-origin Caucasian patients with stable chest pain. Eur. J. Radiol..

[205] Goeller M., Achenbach S., Cadet S., Kwan A.C., Commandeur F., Slomka P.J., Gransar H., Albrecht M.H., Tamarappoo B.K., Berman D.S. (2018). Pericoronary Adipose Tissue Computed Tomography Attenuation and High-Risk Plaque Characteristics in Acute Coronary Syndrome Compared with Stable Coronary Artery Disease. JAMA Cardiol..

[206] Oikonomou E.K., Antonopoulos A.S., Schottlander D., Marwan M., Mathers C., Tomlins P., Siddique M., Klüner L.V., Shirodaria C., Mavrogiannis M.C. (2021). Standardized measurement of coronary inflammation using cardiovascular computed tomography: Integration in clinical care as a prognostic medical device. Cardiovasc. Res..

[207] Oikonomou E.K., Marwan M., Desai M.Y., Mancio J., Alashi A., Hutt Centeno E., Thomas S., Herdman L., Kotanidis C.P., Thomas K.E. (2018). Non-invasive detection of coronary inflammation using computed tomography and prediction of residual cardiovascular risk (the CRISP CT study): A post-hoc analysis of prospective outcome data. Lancet.

[208] Oikonomou E.K., Desai M.Y., Marwan M., Kotanidis C.P., Antonopoulos A.S., Schottlander D., Channon K.M., Neubauer S., Achenbach S., Antoniades C. (2020). Perivascular Fat Attenuation Index Stratifies Cardiac Risk Associated with High-Risk Plaques in the CRISP-CT Study. J. Am. Coll. Cardiol..

[209] Oikonomou E.K., Williams M.C., Kotanidis C.P., Desai M.Y., Marwan M., Antonopoulos A.S., Thomas K.E., Thomas S., Akoumianakis I., Fan L.M. (2019). A novel machine learning-derived radiotranscriptomic signature of perivascular fat improves cardiac risk prediction using coronary CT angiography. Eur. Heart J..

[210] Antoniades C., Antonopoulos A.S., Deanfield J. (2020). Imaging residual inflammatory cardiovascular risk. Eur. Heart J..

[211] Greenland P., Alpert J.S., Beller G.A., Benjamin E.J., Budoff M.J., Fayad Z.A., Foster E., Hlatky M.A., Hodgson J.M., Kushner F.G. (2010). American College of Cardiology Foundation; American Heart Association. 2010 ACCF/AHA guideline for assessment of cardiovascular risk in asymptomatic adults: A report of the American College of Cardiology Foundation/American Heart Association Task Force on Practice Guidelines. J. Am. Coll. Cardiol..

[212] Arnett D.K., Blumenthal R.S., Albert M.A., Buroker A.B., Goldberger Z.D., Hahn E.J., Himmelfarb C.D., Khera A., Lloyd-Jones D., McEvoy J.W. (2019). 2019 ACC/AHA Guideline on the Primary Prevention of Cardiovascular Disease: Executive Summary: A Report of the American College of Cardiology/American Heart Association Task Force on Clinical Practice Guidelines. J. Am. Coll. Cardiol..

[213] Visseren F.L.J., Mach F., Smulders Y.M., Carballo D., Koskinas K.C., Bäck M., Benetos A., Biffi A., Boavida J.M., Capodanno D. (2021). ESC National Cardiac Societies; ESC Scientific Document Group. 2021 ESC Guidelines on cardiovascular disease prevention in clinical practice. Eur. Heart J..

[214] Cosentino F., Grant P.J., Aboyans V., Bailey C.J., Ceriello A., Delgado V., Federici M., Filippatos G., Grobbee D.E., Hansen T.B. (2020). ESC Scientific Document Group. 2019 ESC Guidelines on diabetes, pre-diabetes, and cardiovascular diseases developed in collaboration with the EASD. Eur. Heart J..

[215] Kramer H., Toto R., Peshock R., Cooper R., Victor R. (2005). Association between chronic kidney disease and coronary artery calcification: The Dallas Heart Study. J. Am. Soc. Nephrol..

[216] Gulati M., Levy P.D., Mukherjee D., Amsterdam E., Bhatt D.L., Birtcher K.K., Blankstein R., Boyd J., Bullock-Palmer R.P., Writing Committee Members (2021). 2021 AHA/ACC/ASE/CHEST/SAEM/SCCT/SCMR Guideline for the Evaluation and Diagnosis of Chest Pain: A Report of the American College of Cardiology/American Heart Association Joint Committee on Clinical Practice Guidelines. J. Am. Coll. Cardiol..

[217] Knuuti J., Wijns W., Saraste A., Capodanno D., Barbato E., Funck-Brentano C., Prescott E., Storey R.F., Deaton C., Cuisset T. (2020). ESC Scientific Document Group. 2019 ESC Guidelines for the diagnosis and management of chronic coronary syndromes. Eur. Heart J..

[218] Saraste A., Knuuti J. (2020). ESC 2019 guidelines for the diagnosis and management of chronic coronary syndromes: Recommendations for cardiovascular imaging. Herz.

[219] Narula J., Chandrashekhar Y., Ahmadi A., Abbara S., Berman D.S., Blankstein R., Leipsic J., Newby D., Nicol E.D., Nieman K. (2021). SCCT 2021 Expert Consensus Document on Coronary Computed Tomographic Angiography: A Report of the Society of Cardiovascular Computed Tomography. J. Cardiovasc. Comput. Tomogr..

[220] Maroules C.D., Rybicki F.J., Ghoshhajra B.B., Batlle J.C., Branch K., Chinnaiyan K., Hamilton-Craig C., Hoffmann U., Litt H., Meyersohn N. (2022). 2022 use of coronary computed tomographic angiography for patients presenting with acute chest pain to the emergency department: An expert consensus document of the Society of cardiovascular computed tomography (SCCT): Endorsed by the American College of Radiology (ACR) and North American Society for cardiovascular Imaging (NASCI). J. Cardiovasc. Comput. Tomogr..

[221] Andreini D., Collet C., Leipsic J., Nieman K., Bittencurt M., De Mey J., Buls N., Onuma Y., Mushtaq S., Conte E. (2022). Pre-procedural planning of coronary revascularization by cardiac computed tomography: An expert consensus document of the Society of Cardiovascular Computed Tomography. J. Cardiovasc. Comput. Tomogr..

